# Atomic partial wave meter by attosecond coincidence metrology

**DOI:** 10.1038/s41467-022-32753-8

**Published:** 2022-08-29

**Authors:** Wenyu Jiang, Gregory S. J. Armstrong, Jihong Tong, Yidan Xu, Zitan Zuo, Junjie Qiang, Peifen Lu, Daniel D. A. Clarke, Jakub Benda, Avner Fleischer, Hongcheng Ni, Kiyoshi Ueda, Hugo W. van der Hart, Andrew C. Brown, Xiaochun Gong, Jian Wu

**Affiliations:** 1 State Key Laboratory of Precision Spectroscopy, East China Normal University, Shanghai, China; 2 Centre for Theoretical Atomic, Molecular and Optical Physics, School of Mathematics and Physics, Queen’s University Belfast, University Road, Belfast, BT7 1NN Northern Ireland UK; 3 School of Physics and CRANN Institute, Trinity College Dublin, Dublin 2, Ireland; 4 Institute of Theoretical Physics, Faculty of Mathematics and Physics, Charles University, V Holešovičkách 2, 180 00 Prague 8, Czech Republic; 5 Raymond and Beverly Sackler Faculty of Exact Science, School of Chemistry and Center for Light-Matter Interaction, Tel Aviv University, 6997801 Tel-Aviv, Israel; 6 Collaborative Innovation Center of Extreme Optics, Shanxi University, Taiyuan, Shanxi China; 7 CAS Center for Excellence in Ultra-intense Laser Science, Shanghai, China

**Keywords:** Attosecond science, Ultrafast photonics

## Abstract

Attosecond chronoscopy is central to the understanding of ultrafast electron dynamics in matter from gas to the condensed phase with attosecond temporal resolution. It has, however, not yet been possible to determine the timing of individual partial waves, and steering their contribution has been a substantial challenge. Here, we develop a polarization-skewed attosecond chronoscopy serving as a partial wave meter to reveal the role of each partial wave from the angle-resolved photoionization phase shifts in rare gas atoms. We steer the relative ratio between different partial waves and realize a magnetic-sublevel-resolved atomic phase shift measurement. Our experimental observations are well supported by time-dependent R-matrix numerical simulations and analytical soft-photon approximation analysis. The symmetry-resolved, partial-wave analysis identifies the transition rate and phase shift property in the attosecond photoelectron emission dynamics. Our findings provide critical insights into the ubiquitous attosecond optical timer and the underlying attosecond photoionization dynamics.

## Abstract (web summary)

Understanding the photoelectron emission time after the interaction of photon with atoms and molecules is of fundamental interest. Here the authors examine the role of partial waves to the photoionization phase shift of atoms using an attosecond clock and electron-ion coincidence spectroscopy.

## Introduction

Attosecond light pulses permit real-time observation and precise manipulation of ultrafast electron dynamics^1^. This “attosecond chronoscopy” includes two main approaches: in the frequency domain, the reconstruction of attosecond beating by interference of two-photon transitions (RABBITT) technique^2,3^ and in the time domain, the attosecond streaking camera^4,5^. Both techniques have been applied to a wide range of attosecond time-resolved photoelectron emission dynamics in atoms^6–8^, molecules^9–11^, and condensed matter^12–15^. Studies have demonstrated the photoelectron emission time-delays arising from different ionization shells^16–18^, shape resonances^9,10,19,20^, electron correlation^21^, orbital asymmetry^22^, spatial asymmetry (chirality)^11^, and electron delocalization^23^. These time-delays are attributed to the energy dependence of the scattering phase shift during electron transitions in a short-range potential, Coulomb potential, and the laser-Coulomb-coupling effect in the continuum^24,25^. The scattering phase shift is generally understood in terms of the Eisenbud-Wigner-Smith (EWS) time delay^26,27^, which provides a good description for single-photon ionization. Pioneering reports^6,7^ have demonstrated a good approximation at high photon energies and photoelectron kinetic energies on the basis of the analytical approximation of the continuum-continuum laser-Coulomb-coupling delay (cc-delay)^24,25^. However, at low photoelectron kinetic energies, the photoionization time delay shows a strong dependence on the emission angle^28–30^, and thus the resolution of the magnetic sublevels in the two-photon ionization phase shift^31^, the sensitivity of the attosecond continuum-continuum time delay to the laser field intensity and the ability to steer these dynamics remain topics of interest.

The angular dependence of ionization processes is expressed through the photoelectron angular distribution (PAD), which acts as an interferogram in connecting the experimental observables with the underlying electron wavefunction^32–35^. When the residual singly-charged ion remaining after photoionization contains a single magnetic-sublevel, as is the case for helium, the PAD, *I*(*θ*, *φ*), may be defined as

1 $$I(\theta,\varphi )\propto {\left | \mathop{\sum }\limits_{l=0}^{{l}_{\max }}\mathop{\sum }\limits_{m=-l}^{l}{\beta }_{lm}{Y}_{lm}(\theta,\varphi )\right | }^{2},$$

where the quantization axis is defined by the laser polarization vector, *θ* and *φ* are the polar and azimuthal angles of photoelectron emission direction, *Y*_*lm*_ are the spherical harmonics, and *β*_*lm*_ are expansion coefficients. When multiple residual-ion states are available, the observed PAD is the sum of the individual PADs associated with each residual-ion state. Traditional RABBITT measurements^2,3^ using parallel-polarized extreme ultraviolet attosecond pulse trains (XUV-APT) and near-infrared (NIR) laser fields report a cylindrically symmetric PAD and an angle-dependent phase shift, as large as *π*, which is ascribed to the incomplete quantum interference following Fano’s propensity rule^28,34,36^. However, as the relative polarization direction between the XUV-APT and NIR is skewed^37–39^, the cylindrical symmetry is broken. The induced asymmetry provides an opportunity not only to investigate the two-photon, transition-induced photoionization time-delays, but also to resolve the interference between partial waves with different magnetic quantum numbers.

**Fig. 1:**
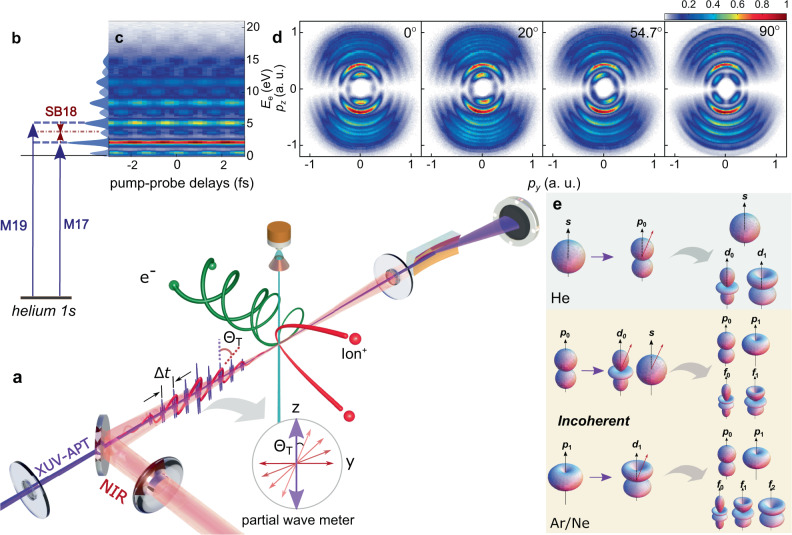
Schematic diagram of attosecond coincidence metrology. **a** Experimental setup. The polarization-skewed XUV-APT and NIR laser field were focused onto a supersonic gas jet via an actively stabilized interferometer. The three-dimensional momenta of the photoionized ions and electrons were measured in coincidence as a function of the XUV-APT/NIR pump-probe delay, and the XUV spectrum is characterized via an online soft-X-ray spectrometer. **b** The principle of sideband generation, and the photoelectron kinetic energy spectrum integrated over photoelectron emission angle and pump-probe delay at Θ_T_ = 0^∘^ in helium. **c** The time-resolved attosecond photoelectron kinetic energy spectrum. **d** Experimentally measured PADs averaged over pump-probe time-delays with a skew-angle of Θ_T_ = 0^∘^, 20^∘^, 54.7^∘^ and 90^∘^, respectively. **e** The two-photon quantum transition maps of helium, neon, and argon.

In this work, we employ an advanced attosecond coincidence metrology, where the polarization axes of the XUV-APT and NIR laser pulses are rotated from a parallel to a perpendicular orientation, as illustrated in Fig. 1a, to serve as a partial-wave meter. This polarization-skewed RABBITT scheme allows us to monitor the photoelectron emission dynamics in time and space simultaneously by steering the different partial waves in the final sideband electrons (see the inset of Fig. 1a). PADs of helium, neon, and argon atoms are measured with an attosecond time-delay axis to reveal the role of symmetry and scattering phase at the instant of photoionization. Significant cylindrical asymmetry is observed both in the PADs and atomic phase shifts as a function of the photoelectron emission angle, *θ*, and polarization skew angle, Θ_T_. The skewed PADs and asymmetric phase shift distribution arise from the coherent interference between different partial waves and incoherent sum between different ionic degenerate channels. Ab initio theoretical simulations agree well with the experimental observations and facilitate the resolution of the two-photon transition phase shifts for each partial wave. Symmetry-resolved partial-wave analysis demonstrates that each partial wave presents a homogeneous scattering phase shift distribution. The *m*-resolved partial-wave phase shifts have been reconstructed both from the experimental measurements and the theoretical simulations. In neon and argon, the skew-angle Θ_T_ provides an attosecond photoemission time-delay manipulation dial by modifying the contribution of different *m*-values of the outgoing electrons within the (even-harmonic) sidebands.

## Results

### Attosecond coincidence interferometer

Our experiment employs an advanced attosecond coincidence interferometer constructed by combining the RABBITT attosecond clock^2^ and electron-ion three-dimensional momentum coincidence spectroscopy^40,41^. The linearly polarized NIR field dresses the continuum electron wavepacket with the joint XUV-APT via a two-photon process, as illustrated in Fig. 1a. The XUV-APT, produced via high harmonic generation in an argon gas filled cartridge^42,43^, contains from harmonic order 13 (20.4 eV) to 25 (39.3 eV) releasing the main photoelectron bands from M13 to M25 in argon (${I}_{{{{{{{{\rm{p}}}}}}}}}^{{{{{{{{\rm{Ar}}}}}}}}}$ = 15.76 eV), M15 to M25 in neon (${I}_{{{{{{{{\rm{p}}}}}}}}}^{{{{{{{{\rm{Ne}}}}}}}}}$ = 21.56 eV), and M17 to M25 in helium (${I}_{{{{{{{{\rm{p}}}}}}}}}^{{{{{{{{\rm{He}}}}}}}}}$ = 24.59 eV). Absorption of an XUV photon of harmonic order 2*n* + 1 or 2*n* − 1 is followed by the absorption or emission of a NIR photon. Thus, the same final electron energy is accessible by two distinct, two-photon pathways. Figure 1b shows the photoelectron kinetic energy distribution in helium with the skew-angle, Θ_T_ = 0^∘^, including the sidebands of SB16 to SB24. Figure 1c shows the measured photoelectron spectra as a function of the relative pump-probe time delay, and Fig. 1d shows the measured PADs of helium under different skewed polarizations between XUV-APT and NIR with Θ_T_ = 0^∘^, 20^∘^, 54.7^∘^, and 90^∘^, respectively. PADs measured for neon and argon are provided in the supplementary information (SI), and are compared to PADs calculated using the R-matrix with time-dependence (RMT) code^44^. Owing to the absorption and emission of a NIR photon from the neighboring main bands, the initial oscillation phase of the sidebands encodes the chirp difference between the neighboring harmonic combs of the XUV-APT, and the photoionization electron transition and propagation phase shifts of interest as compared to a free electron wavepacket with the same kinetic energy in the continuum. The sideband oscillation can be expressed as a function of the pump-probe time delay of *τ*, as $S(\tau,\theta )\propto {A}_{2\omega }\cos (2{\omega }_{{{{{{{{\rm{NIR}}}}}}}}}\tau+{\phi }_{0}(\theta ))$, where *ω*_NIR_ is the NIR frequency and *θ* is the emission angle of the photoelectrons in the polarization plane. The sideband oscillation phase term *ϕ*_0_(*θ*) can be further decomposed into a sum of the atomic phase shift and XUV chirp as *ϕ*_0_(*θ*) = *ϕ*^2*hν*^(*θ*) + *ϕ*_XUV-APT_.

**Fig. 2:**
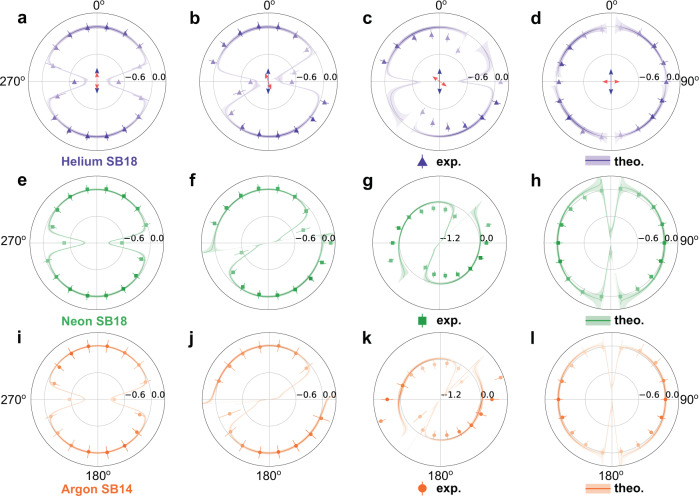
Angle-resolved atomic relative phase shift. **a**–**d** The normalized atomic phase shift distributions, ${{\Delta }}{\phi }_{{{{{{{{\rm{rel}}}}}}}}}^{{{{\Theta }}}_{{{{{{{{\rm{T}}}}}}}}}}(\theta )$, in units of *π* radians as a function of the photoelectron emission angle of helium at a skew-angle of Θ_T_ = (**a**) 0^∘^, (**b**) 20^∘^, (**c**) 54. 7^∘^, and (**d**) 90^∘^. The triangles and solid lines show the experimental and theoretical results, respectively. The error bars in the experimental results represent the standard deviation. The shaded area indicates the fitting error uncertainty and the line colors are weighted by the yield of the photoelectron angular distribution. **e**–**h**, **i**–**l** As **a**–**d** but for the SB18 of neon and SB14 of argon, respectively.

To illustrate the angle-resolved phase shifts, we define a normalized phase shift term as ${{\Delta }}{\phi }_{{{{{{{{\rm{rel}}}}}}}}}^{{{{\Theta }}}_{{{{{{{{\rm{T}}}}}}}}}}(\theta )={\phi }_{0}(\theta )-{\phi }_{0}({{{\Theta }}}_{{{{{{{{\rm{T}}}}}}}}})$ to cancel out the XUV chirp. Figure 2 illustrates comparisons between the experimental observations and our full quantum simulations, calculated using the RMT code. The simulations agree well with the experiments. Figure 2a–d shows ${{\Delta }}{\phi }_{{{{{{{{\rm{rel}}}}}}}}}^{{{{\Theta }}}_{{{{{{{{\rm{T}}}}}}}}}}(\theta )$ of SB18 in helium with Θ_T_ = 0^∘^, 20^∘^, 54.7^∘^, and 90^∘^, respectively. To highlight the regions where the phase is well defined, the relative strength of the line colors is weighted by the photoelectron yield as a function of *θ*. Figure 2e–h and i–l show the ${{\Delta }}{\phi }_{{{{{{{{\rm{rel}}}}}}}}}^{{{{\Theta }}}_{{{{{{{{\rm{T}}}}}}}}}}(\theta )$ of SB18 (6.7 eV) in neon and SB14 (6.2 eV) in argon. The similarity in electron kinetic energy allows us to compare these atomic phase shifts directly. The phase shift distributions at Θ_T_ = 0^∘^ in Fig. 2a, e, i show a cylindrical symmetry, and the maximum variation of ${{\Delta }}{\phi }_{{{{{{{{\rm{rel}}}}}}}}}^{{{{\Theta }}}_{{{{{{{{\rm{T}}}}}}}}}}$ drift to − 0.92*π* (helium), − 0.89*π* (neon), and − 0.93*π* (argon) along the photoemission direction perpendicular to the NIR polarization axis. Here, the photoelectron yield is extremely weak, and the phase jump of approximately *π* radians corresponds to the singularity where the cross section goes through zero. The simulated PADs in Fig. 2 continue to show *C*_2_ rotational symmetry as Θ_T_ is skewed to an arbitrary angle, Θ_T_ ≠ 0 or 90^∘^, but the plane mirror symmetry is broken. At Θ_T_ = 54. 7^∘^, the PAD of helium shows a fourfold distribution and a significant photoelectron yield perpendicular to the NIR polarization axis. The relative atomic phase shift, ${{\Delta }}{\phi }_{{{{{{{{\rm{rel}}}}}}}}}^{54.{7}^{\circ }}$ displays an analogous fourfold angle dependence. For Θ_T_ = 90^∘^, the phase is independent of the photoelectron emission angle, giving a homogeneous distribution in Fig. 2d.

### Symmetry-resolved photoionization time-delays in helium atoms

**Fig. 3:**
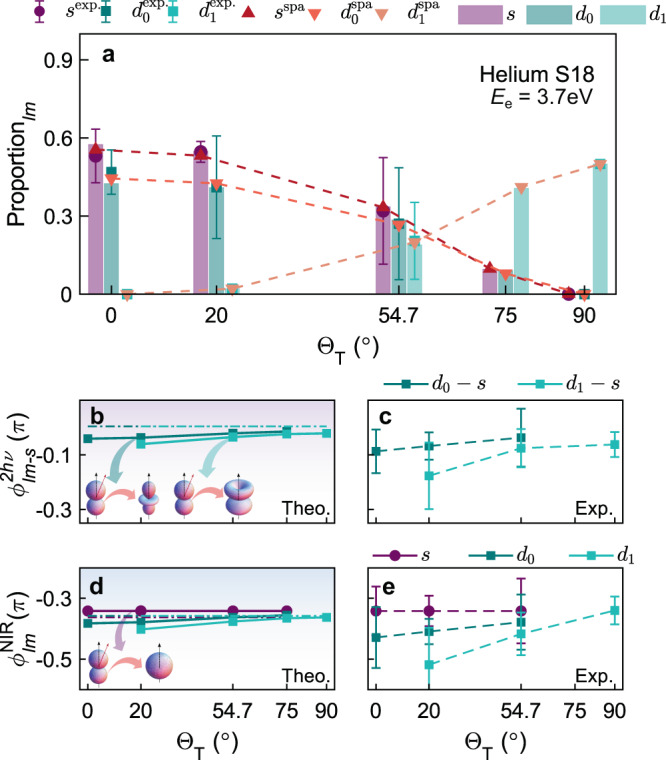
Full partial-wave analysis of helium. **a** The proportions of *m*-resolved partial waves averaged over all pump-probe time-delays as a function of the skew-angle Θ_T_. The purple circles, dark-cyan and turquoise squares show the complex fitting results from experiments of *s*, *d*_0_- and *d*_1_-wave, and the bars with the same color display the RMT simulation. The red triangles represent the SPA predictions. **b**, **c** The *m*-resolved phase shift difference between *d*-waves and *s*-wave from (**b**) theory and (**c**) experiment. The dot-dashed lines display the results with *I*_NIR_ = 0.1TW/cm^2^. **d**, **e** The *m*-resolved continuum-continuum phase shifts from **d** theory and **e** experiment. The error bars represent the standard deviation. The insets show the quantum transition pathways along **b** *p*_0_ → *d*_0_ or *d*_1_, **d** *p*_0_ → *s*. Owing to the identical phase property between *d*_+1_ and *d*_−1_ wave in our RMT simulation, we use *d*_1_ to refer to both the *d*_±1_-waves.

For He, we can perform a full, partial-wave analysis of the experimental and theoretical results, shown in Fig. 3, to understand the behavior of the observed phase shift steered by the NIR fields. Figure 3a presents the relative proportion of *s*-, *d*_0_-, and *d*_±1_-waves as a function of the skew-angle Θ_T_. In the case of Θ_T_ = 0^∘^, only *d*_0_ and *s* partial waves are allowed following the two-photon ionization. As Θ_T_ increases to 90^∘^, the yield of *s* and *d*_0_ partial waves drops to zero, leading to the pure *d*_±1_ waves in the limit of Θ_T_ = 90^∘^. Since the partial-*m*-waves have the same angular characteristics and phase shifts for positive and negative *m*, the homogeneous phase shift distribution of the *d*_±1_ partial wave provides direct evidence of the constant phase shift of each partial wave during the XUV and NIR photon transition (See SI for more details). To provide an analytical expression for the Θ_T_ dependence of the partial-wave ratios, we employ the “soft-photon approximation” (SPA)^45^. It states that the *S*-matrix transition amplitude for a coherent two-photon RABBITT ionization processes takes the form

2 $${S}^{\pm 1} \, \sim \, {J}_{\mp }({{{{{{{{\boldsymbol{\alpha }}}}}}}}}_{{{{{{{{\rm{NIR}}}}}}}}}\cdot {{{{{{{{\bf{k}}}}}}}}}_{\pm }){e}^{-i({\phi }_{{{{{{{{\rm{XUV}}}}}}}}}\;\pm {\phi }_{{{{{{{{\rm{NIR}}}}}}}}}\;)}\left\langle {\chi }_{{{{{{{{{\bf{k}}}}}}}}}_{\pm }}|{{{{{{{{\boldsymbol{\epsilon }}}}}}}}}_{{{{{{{{\rm{XUV}}}}}}}}}\cdot {{{{{{{\bf{p}}}}}}}}|{{{\Psi }}}_{{{{{{{{\rm{g}}}}}}}}}\right\rangle,$$

where + ( − ) denotes the absorption (emission) process, *J*_*n*_ is a Bessel function of the first kind, ***α***_NIR_ and ***ϵ***_XUV_ are the polarization vectors of the NIR and XUV pulses, *ϕ*_XUV_ and *ϕ*_NIR_ are the carrier-envelope phases of the XUV and NIR pulses, respectively, **k**_±_ is the photoelectron momentum at the sideband, **p** is the momentum operator, and Ψ_g_ and *χ*_**k**_ are the ground-state and final continuum-state wavefunctions. The advantage of this expression is that it allows the dependence on Θ_T_ to be made explicit through the polarization vector ***α***_NIR_. In the case of helium, the *S*-matrix transition amplitude for two-photon ionization is given by

3 $${S}^{\pm 1} \sim 	\cos {{{\Theta }}}_{{{{{{{{\rm{T}}}}}}}}}\left[\frac{1}{3}{Y}_{00}({\theta }_{k},{\varphi }_{k})+\frac{2}{\sqrt{45}}{Y}_{20}({\theta }_{k},{\varphi }_{k})\right] \\ 	+\sin {{{\Theta }}}_{{{{{{{{\rm{T}}}}}}}}}\ i\sqrt{\frac{1}{30}}\left[{Y}_{21}({\theta }_{k},{\varphi }_{k})+{Y}_{2-1}({\theta }_{k},{\varphi }_{k})\right],$$

The normalized yield of each partial wave as a function of Θ_T_ is given by ${P}_{s}\,\sim \frac{1}{9}{\cos }^{2}{{{\Theta }}}_{{{{{{{{\rm{T}}}}}}}}}$, ${P}_{{d}_{0}} \sim \frac{4}{45}{\cos }^{2}{{{\Theta }}}_{{{{{{{{\rm{T}}}}}}}}}$, and ${P}_{{d}_{\pm 1}} \sim \frac{1}{30}{\sin }^{2}{{{\Theta }}}_{{{{{{{{\rm{T}}}}}}}}}$. As shown in Fig. 3a, the SPA results accurately predict the weights of the different partial waves.

The phase shift for each partial wave is obtained within the RMT calculations by considering PADs, which include only that specific partial wave. To extract the *m*-resolved phase shifts from the experimental measurements, we performed a complex fitting of the argument and absolute square of the partial-wave interference, ${{{{{{{{\rm{SB}}}}}}}}}_{{{{{{{{\rm{He}}}}}}}}}(\theta,\varphi )={\sum }_{l,m}{c}_{lm}{Y}_{lm}(\theta,\varphi ){e}^{-i{\phi }_{lm}^{2h\nu }}$, to the measured ${{\Delta }}{\phi }_{{{{{{{{\rm{rel}}}}}}}}}^{{{{\Theta }}}_{{{{{{{{\rm{T}}}}}}}}}}(\theta )$ distributions in Fig. 2a–d and the PADs, where *c*_*lm*_ is the amplitude of the partial wave and ${\phi }_{lm}^{2h\nu }$ is the two-photon transition phase shift. The relative ratios and the *m*-resolved partial-wave phase shifts, ${\phi }_{lm}^{2h\nu }$, can be reconstructed from this fitting procedure (see methods for more details). Figure 3b, c present the reconstructed phase shift of the *d*_0_ and *d*_±1_ partial waves with respect to the *s*-wave, as defined by ${\phi }_{lm-s}^{2h\nu }={\phi }_{lm}^{2h\nu }-{\phi }_{s}^{2h\nu }$. The directly-computed, RMT results agree well with the reconstructed, experimental results. The phase shift difference between the *d*_0,±1_ and *s* waves is around 0 with a maximum deviation around 0.04*π* for the *d*_0_-wave at Θ_T_ = 0^∘^, and 0.06*π* for the *d*_±1_-wave at Θ_T_ = 20^∘^. Here, the partial-wave phase shift includes two main components, ${\phi }_{lm}^{2h\nu }={\phi }_{lm}^{{{{{{{{\rm{EWS}}}}}}}}}+{\phi }_{lm}^{{{{{{{{\rm{NIR}}}}}}}}}$, where ${\phi }_{lm}^{{{{{{{{\rm{EWS}}}}}}}}}$ is the one-photon scattering phase shift^46^ during the transition of *s* → *p*_0_—i.e., the EWS time delay in the time domain^26,27^—and ${\phi }_{lm}^{{{{{{{{\rm{NIR}}}}}}}}}$ is the continuum-continuum phase shift following the transitions of *p*_0_ → *s*, or *p*_0_ → *d*_0_ and *d*_±1_ induced by NIR photon absorption or emission in the long-range Coulomb potential of the ionic core^24^. Since all partial waves in the measured sideband arise from the same intermediate *p*_0_ state, the observed skew-angle dependence of the partial-wave phase shift must originate from the continuum-continuum transition process.

Figure 3b also shows the *m*-resolved relative partial-wave phase shift as a function of Θ_T_ with an extremely weak NIR pulse (of intensity 0.1 TW/cm^2^, dot-dashed lines) in the perturbative regime. All partial waves present a constant phase shift over all skew angles: ${\phi }_{s}^{2h\nu }=-0.109\pi$ and ${\phi }_{{d}_{0,\pm 1}}^{2h\nu }=-0.104\pi$. This indicates that when the NIR field is above the low intensity limit, as it rotates from parallel to perpendicular orientation, the effective laser field intensity projected in the polarization direction of each partial wave decreases, giving rise to a positive phase shift. This field dependence is negligible in the *s*-wave because of its isotropic character. On the basis of the calculated EWS time delay (or phase shift) of helium, ${\phi }_{s\to {p}_{0}}^{{{{{{{{\rm{EWS}}}}}}}}}$, we may further determine the absolute continuum-continuum phase shift, ${\phi }_{lm}^{{{{{{{{\rm{NIR}}}}}}}}}$ = ${\phi }_{lm}^{2h\nu }$ – ${\phi }_{s\to {p}_{0}}^{{{{{{{{\rm{EWS}}}}}}}}}$, as a function of Θ_T_, see Fig. 3d (RMT simulations), and Fig. 3e (experimentally reconstructed results).

### Partial-wave decomposition for neon and argon

The relative atomic phase shift ${{\Delta }}{\phi }_{{{{{{{{\rm{rel}}}}}}}}}^{{{{\Theta }}}_{{{{{{{{\rm{T}}}}}}}}}}(\theta )$ of neon and argon in Fig. 2 show a two-fold angle dependence from Θ_T_ = 0^∘^ to 90^∘^ with the same cylindrical and rotation symmetry as in helium. Unlike helium’s simple, *s* symmetry, the outermost electron in neon and argon can have both *p*_0_ and *p*_±1_ character, coupled to residual-ion states of *P*_0_ and *P*_∓1_ symmetry, respectively. Thus, the two-photon ionization dynamics are significantly more complicated, and the final PAD observed is the incoherent sum over the ${p}_{0}\to (s\,{{\mbox{or}}}\,{d}_{0})\to \left(\,{f}_{0,\pm 1}\,{{\mbox{or}}}\,{p}_{0,\pm 1}\right)$ and ${p}_{\pm 1}\to {d}_{\pm 1}\to \left(\,{f}_{0,\pm 1,\pm 2}\,{{\mbox{or}}}\,{p}_{0,\pm 1}\right)$ pathways, as illustrated in Fig. 1e.

**Fig. 4:**
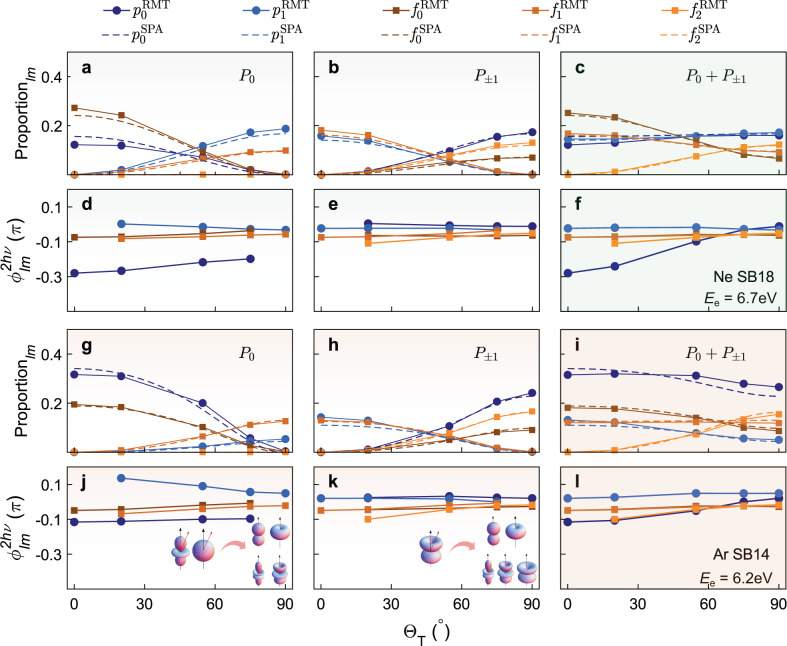
Full partial-wave analysis of neon and argon. **a**–**c** Partial-wave proportions (averaged over all pump-probe time-delays) as a function of Θ_T_ in neon for the residual ionic states of **a** *P*_0_, **b** *P*_±1_, and **c** the incoherent sum of *P*_0_ and *P*_±1_. **d**–**f** The *m*-resolved two-photon phase shifts in neon for the same initial states as **a**–**c**. The RMT calculated proportions and phase shifts are labeled as solid dot lines, *p*_0_ (dark blue), *p*_1_ (light blue), *f*_0_ (dark orange), *f*_1_ (orange), and *f*_2_ (light orange). The SPA predictions are labeled as dashed lines with the same coloring as RMT. **g**–**l** As **a**–**f** but for argon.

Figure 4a, d show the simulated ionic-state-resolved partial-wave proportions and phase shifts as a function of skew-angle Θ_T_ of neon with the *P*_0_ residual-ion state. The variation in the relative proportion of *f*-waves is analogous to the *d*-waves in helium in that the yield of the *f*_0_-wave is proportional to ${\cos }^{2}{{{\Theta }}}_{{{{{{{{\rm{T}}}}}}}}}$, and the yields of *f*_±1_-waves are proportional to ${\sin }^{2}{{{\Theta }}}_{{{{{{{{\rm{T}}}}}}}}}$ (see SI for details of the SPA expressions). Only *p*_0_ and *f*_0_ partial waves contribute at Θ_T_ = 0^∘^, and only *p*_±1_ and *f*_±1_ waves at Θ_T_ = 90^∘^. The phase shift of the *p*_0_-wave increases by 0.082*π* as Θ_T_ changes from 0^∘^ to 75^∘^. However, the phase shift of the *p*_±1_-wave, ${\phi }_{{p}_{\pm 1}}^{2h\nu }$, decreases by 0.034*π* from 20^∘^ to 90^∘^. Considering the angular dependence of the individual partial waves, the NIR field aligns with the *p*_0_ wave for a skew-angle of 0^∘^, and with the *p*_±1_ wave for a skew-angle of 90^∘^. This gives rise to the opposite dependence of ${\phi }_{lm}^{{{{{{{{\rm{NIR}}}}}}}}}$ on skew-angle between *p*_0_ and *p*_±1_. These small variations in the partial-wave phase shifts disappear at the low NIR-intensity limit as shown in the He case. The phase shifts of the *f*-waves increase slightly by 0.038*π* and 0.025*π* for *f*_0_- and *f*_±1_-waves, respectively. Figure 4b, e demonstrate the simulated *m*-resolved relative proportions and partial-wave phase shifts with the *P*_±1_ residual-ion states. One-photon ionization creates only the *d*_∓1_-wave, thus the projection angle into the rotated coordinate system is complementary to the the case with the *P*_0_ symmetry. Also in contrast to *P*_0_, the sideband electron only includes *p*_∓1_-, *f*_∓1_-waves at 0^∘^ and *p*_0_-, *f*_0_- and *f*_∓2_- waves at 90^∘^. The phase shifts of *p*_0_ and *p*_±1_ display a more subtle variation but ${\phi }_{{p}_{0}}^{2h\nu }$ averaged over the skew-angle is shifted by 0.23*π* compared to the *P*_0_ case. Similar behavior is observed for *f*_±1,±2_ in that ${\phi }_{{f}_{\pm 1}}^{2h\nu }$ and ${\phi }_{{f}_{\pm 2}}^{2h\nu }$ increase by 0.038*π* and 0.058*π*, respectively, as the NIR field is skewed away from the XUV-APT while ${\phi }_{{f}_{0}}^{2h\nu }$ remains approximately constant.

As shown in Fig. 4c, f, the total *m*-resolved partial-wave proportions and phase shifts arise from the incoherent sum over the individual *P*_0_ and *P*_±1_ ionic states. As Θ_T_ rotates from 0^∘^ to 90^∘^, the total partial-wave phase of *p*_±1_, *f*_0_, *f*_±1_-waves are approximately constant, and the ${\phi }_{{f}_{\pm 2}}^{2h\nu }$ phases present a subtle variation over 0.058*π*, since *f*_±2_ final electronic states can only be populated with the *P*_∓1_ residual-ion state. However, the ${\phi }_{{p}_{0}}^{2h\nu }$ phase in neon can be steered from − 0.28*π* to − 0.01*π* via the manipulation of the relative proportion of *p*_0_(*P*_0_) and *p*_0_(*P*_±1_), which provides a knob to continually steer the photoemission time delay over 177 attoseconds in an extremely high attosecond time resolution.

Figure 4g–l show the simulated *lm*-resolved partial-wave ionization yields and two-photon transition phase shifts of argon with the same ionic states as Fig. 4a–f. The skew-angle-induced change in the proportion of each partial-wave follows the same rule as in neon but shows differences in the relative ratios of *p*- and *f*-waves. The most significant difference is that the proportion of the *p*_0_-wave is larger than the *f*_0_-wave in argon with both the *P*_0_ and *P*_±1_ residual-ion states. By contrast, in neon the *f*_0_-wave dominates for the *P*_0_ state but the *p*_0_-wave dominates for *P*_±1_. Also the ratio *p*_±1_: *f*_±1_ shows slight differences between neon and argon for both *P*_0_ and *P*_±1_ residual-ion states. Since the final *p*-waves are produced via *s* and *d* intermediate states, this discrepancy is mainly from the different relative populations of the intermediate states. For the partial-wave phase shifts in argon, besides the same skew-angle dependence for each partial wave as shown in neon, each ${\phi }_{lm}^{2h\nu }$ in argon is larger than in neon, especially for the *p*_0_-wave coupled to the *P*_0_ residual-ion state. This character is mainly attributed to the smaller value of the short-range phase *δ*_2*p*→*s*_ in neon during the ionization from the initial 2*p* state to the *s* state, which is included in the EWS phase term^47^.

### Two-photon phase shift difference between *p* and *f* partial waves

**Fig. 5:**
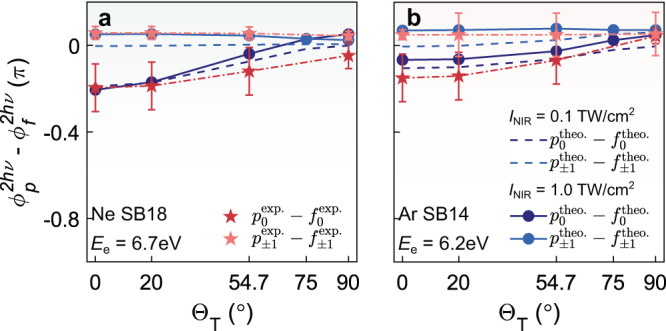
Relative partial-wave-resolved phase shift between *p*- and *f*-waves. **a** The skew-angle-dependent two-photon transition phase shift between *p*_0_/*f*_0_ and *p*_±1_/*f*_±1_ in neon atoms at SB18. The dark and light red stars plot the reconstructed ${{\Delta }}{\phi }_{{p}_{0}-{f}_{0}}^{2h\nu }$, ${{\Delta }}{\phi }_{{p}_{1}-{f}_{1}}^{2h\nu }$ from the experimental results and the error bars represent the standard deviation. The blue circles and dashed lines show the theoretically simulated results from the RMT simulation under the NIR intensity of *I*_NIR_ = 1.0TW/cm^2^ and *I*_NIR_ = 0.1TW/cm^2^, respectively. **b** As **a** but for the argon atom.

We may reconstruct the relative two-photon transition phase shift between *p*- and *f*-waves for neon and argon using the same procedure as for the *s* and *d* waves in helium. However, the different residual-ion states, *P*_0_ and *P*_±1_, are not resolved in the experiment, and thus we cannot extract the individual partial-wave proportions. We compensate for this by using the proportions as predicted by the RMT simulation (SPA in SI) in the fitting procedure for the experimental PADs, allowing us to compute the relative phase shift for partial waves with the same magnetic quantum number—${{\Delta }}{\phi }_{{p}_{0}-{f}_{0}}^{2h\nu }$ and ${{\Delta }}{\phi }_{{p}_{\pm 1}-{f}_{\pm 1}}^{2h\nu }$, as shown in Fig. 5. The extracted ${{\Delta }}{\phi }_{{p}_{m}-{f}_{m}}^{2h\nu }$ agree well with the RMT simulations, and it demonstrates a positive phase variation trend as a function of skew-angle, which is attributed to the relative ratio variations of photoelectrons coupled to different residual ionic states.

In this picture then, we divide the electron transition process into two steps. After the initial XUV-APT ionization, the skewed NIR field introduces a new coordinate system, rotated by an angle Θ_T_ with respect to the original frame. Therefore, the one-photon, continuum photoelectron wavepacket, whether absorbing or emitting one NIR photon, should include full partial-wave interference after projection onto the new rotated coordinates. Although the soft-photon approximation is able to predict the weights of the different partial waves reasonably accurately, as shown in Fig. 4, it predicts phase shifts, which depend only on the orbital angular momentum *l*, and not on the magnetic quantum number *m*. Advanced approaches, such as RMT, are required to illustrate the NIR-intensity dependence in the continuum-continuum transition delays. These phase shifts can be associated with subtle changes in the structure of the bound and continuum atomic states due to the XUV and NIR laser fields, and can form the basis for new types of exploration of atomic and molecular properties in ultrafast laser fields.

## Discussion

In summary, we employed an advanced attosecond coincidence metrology to investigate the atomic phase shift induced by two-photon ionization as a function of the photoelectron emission angle in helium, neon and argon. By controlling the relative polarization angle between the XUV-APT and NIR laser pulses, we observe a phase shift close to *π* in the photoelectron emission direction perpendicular to the NIR polarization axis. The phase shifts show a skew-angle dependence, which disappears at low NIR-intensity, and the deviations scale with the square of the intensity. Thus, the observed behavior may be attributed to the interaction of the NIR with the outgoing electron beyond lowest-order perturbation theory. Our quantum RMT calculations and semi-classical soft-photon approximation model agree well with our experimental observations, further demonstrating that the angular variation in phase shifts originates from the quantum interference between magnetic-sublevel-resolved partial waves and the incoherent sum over residual ionic states. A magnetic-sublevel-resolved partial-wave meter is demonstrated in helium both experimentally and theoretically, and is extended into the initial ionic states-resolved atomic phase shifts in neon and argon. The observations also illustrate that the continuum-continuum phase shift can be precisely manipulated by tuning the skew-angle and NIR intensity.

Our findings provide critical insights into the attosecond atomic clock and the underlying time-resolved photoionization dynamics, and also open a pertinent question related to photoionization time-delays with skewed polarization or circular polarization. How do the sophisticated two-photon transition elements relate to symmetry breaking in the full atomic system, including the laser field coupling in a long-range Coulomb potential, transitions involving the continuum electron, and the accurate atomic potential, which is polarized by the NIR field? Additionally, our experimental methods provide a state-of-the-art approach, which can investigate and manipulate symmetry-resolved photoemission dynamics on an attosecond time scale, including shape resonances dominated by high-angular-momentum continuum waves^9,10,20^, constructive and destructive interference between multiple coupled states^48^, *m*-resolved transition dynamics^31^, photoelectron circular dichroism^49–51^, and correlated shake-up and shake-off double ionization assisted by Auger decay^18,21^.

## Methods

### Attosecond coincidence interferometer

The attosecond photoelectron spectra are measured via an attosecond coincidence interferometer^20,23^. A multipass amplified Titanium-Sapphire laser system that produces a near-infrared (NIR) femtosecond laser pulse with a central wavelength of 790 nm, 1.4 mJ at 10 kHz repetition rate and a pulse duration of 28 fs (full-width at half-maximum in intensity), is used to construct our attosecond optical interferometer through a nonlinear Mach-Zehnder interferometer. The XUV-APT is generated by focusing the NIR pulse into a capillary filled with argon gas. The generated XUV spectrum is filtered through a coaxial aluminum foil of thickness 200 nm installed on a quartz ring, yielding a final photon energy range from 13th order (H13, 20.4 eV) to 25th order (H25, 39.3 eV). The filtered XUV-APT is recombined with the dressing NIR pulse through a central hole siliver mirror. The XUV-APT and NIR laser fields are phase-locked in the time domain and their relative time delay is manipulated via a delay stage with an attosecond time resolution. To achieve an attosecond stability, the nonlinear interferometer is actively stabilized^17,20,23,52^ with a time-jitter below 40 as. The delay-controlled XUV-APT and NIR were focused onto the supersonic gas jet. The single photoionization induced ion and electron fragments were guided by a homogeneous electric and magnetic field towards the ion and electron detectors. Their three-dimensional momenta were reconstructed from the positions and times of flight recorded from the detectors, which contain two micro-channel plates (MCP) and delayline anodes^53,54^. To investigate the complete, angle-resolved photoelectron emission phase shifts, all ionized electrons are accumulated in a single recording window to achieve a 4*π* solid angle detection, and the released photoelectrons are measured as a function of the relative time delay between the phase-locked XUV-APT and NIR pulse, where the positive time delay means that the XUV-APT arrives after the NIR pulse. The intensity of the NIR laser field in the interaction range is estimated to be 1 × 10^12^ W/cm^2^^55^.

### Ab initio simulations

Theoretical results are obtained using the R-matrix with time-dependence (RMT) code^44,56,57^. The method employs the R-matrix division of space, whereby a small (20 *a*_0_), inner region contains the He^+^/Ar^+^/Ne^+^ ion, coupled to a continuum electron. In this region, full account is taken of electron exchange. In the outer region, a single ionized electron can propagate far from the residual ion, and we neglect exchange involving this electron. This permits the tractable solution of the time-dependent Schrödinger equation in each region, and the wavefunction is matched at the boundary. The description of argon and neon used in the calculations follows ref. 58, and helium follows the so-called “1T” model described in ref. 59.

To model the RABBITT measurements, the time-varying electric field is treated classically within the dipole approximation. The NIR-pulse is modeled as a 10-cycle, 760 nm pulse, with a peak intensity of 10^12^ W/cm^2^ and 3 cycle ${\sin }^{4}$ ramp on/off. This equates to a pulse duration of 25 fs. The XUV-APT has a peak intensity of 10^10^ W/cm^2^ and is constructed to approximate the frequency comb of the experimental XUV-APT. We propagate the wavefunction for a further 12 fs after the end of the pulse, for a total propagation time of 36 fs, allowing the outgoing wavepacket to be resolved from the bound-state wavefunction. The ejected electron is described up to a distance of 5160 *a*_0_ from the nucleus. We calculate the final wavefunction for 16 time-delays spanning a single cycle of the NIR field.

Following time propagation, we obtain the photoelectron momentum distribution in the laser polarization ( *y* − *z*) plane, by decoupling the photoelectron wavefunction from that of the residual ion, and transforming into momentum space via a Fourier Transform. This momentum distribution is resolved into the individual partial-wave contributions by selecting the requisite *l* and *m* of the outgoing electron. The *lm*-resolved yields are then obtained by integrating these distributions over the sideband momentum and angular variables.

### Analytical analysis of two-photon ionization time-delays

The analytical dependence of the ionization yield on relative polarization angle can be obtained using the “soft-photon approximation”^45^, which provides an expression for the *S*-matrix transition amplitude for two-photon XUV-NIR processes. The method is based on the strong-field approximation, and the variation with relative polarization angle is extracted through a first-order, time-dependent perturbation approach. Further details on the expressions for the *m*-resolved photoelectron yields are provided in the SI.

### Partial wave-resolved complex fitting of interference phase

The single-photon ionization of helium from the 1*s*^2^ ground-state gives a *p*_0_ continuum wavepacket, and the later absorption or emission of one NIR photon creates final continuum wavepacket from the coherent sum of *s*- and *d*_0,±1_-waves. Thus, the experimentally measured photoelectron angular distributions (PADs) can be described by:

4 $${I}_{{{{{{{{\rm{e}}}}}}}}}^{{{{{{{{\rm{He}}}}}}}}}(\theta,\varphi ) \sim 	\left | \left[{c}_{s}Y_{00}(\theta,\varphi ){e}^{-i{\phi }_{s}^{2h\nu }}+{c}_{{d}_{0}}Y_{20}(\theta,\varphi ){e}^{-i{\phi }_{{d}_{0}}^{2h\nu }}\right]\cos {{{\Theta }}}_{{{{{{{{\rm{T}}}}}}}}}\right.\\ 	 {\left.+i{c}_{{d}_{1}}\left[{Y}_{21}(\theta,\varphi ){e}^{-i{\phi }_{{d}_{1}}^{2h\nu }}+{Y}_{2-1}(\theta,\varphi ){e}^{-i{\phi }_{{d}_{-1}}^{2h\nu }}\right]\sin {{{\Theta }}}_{{{{{{{{\rm{T}}}}}}}}}\right | }^{2}.$$

where ${c}_{s}\cos {{{\Theta }}}_{{{{{{{{\rm{T}}}}}}}}},{c}_{{d}_{0}}\cos {{{\Theta }}}_{{{{{{{{\rm{T}}}}}}}}},{c}_{{d}_{1}}\sin {{{\Theta }}}_{{{{{{{{\rm{T}}}}}}}}}$ are the amplitudes of each partial wave and ${\phi }_{s}^{2h\nu },{\phi }_{{d}_{0}}^{2h\nu },{\phi }_{{d}_{\pm 1}}^{2h\nu }$ are the corresponding RABBITT phases. The emission angle-resolved atomic phase is:

5 $${\phi }_{{{{{{{{\rm{He}}}}}}}}}^{2h\nu }(\theta,\varphi ) \sim 	\arg \left\{\left[{c}_{s}Y_{00}(\theta,\varphi ){e}^{-i{\phi }_{s}^{2h\nu }}+{c}_{{d}_{0}}Y_{20}(\theta,\varphi ){e}^{-i{\phi }_{{d}_{0}}^{2h\nu }}\right]\cos {{{\Theta }}}_{{{{{{{{\rm{T}}}}}}}}}\right.\\ 	 \left.+i{c}_{{d}_{1}}\left[{Y}_{21}(\theta,\varphi ){e}^{-i{\phi }_{{d}_{1}}^{2h\nu }}+{Y}_{2-1}(\theta,\varphi ){e}^{-i{\phi }_{{d}_{-1}}^{2h\nu }}\right]\sin {{{\Theta }}}_{{{{{{{{\rm{T}}}}}}}}}\right\}.$$

We use Eq. (4) and Eq. (5) to fit the experimentally measured PADs and relative phase shift distributions as a function of photoelectron emission angle *θ* to reconstruct the partial-wave information, where the constraints of the parameters are $|{c}_{s}\cos {{{\Theta }}}_{{{{{{{{\rm{T}}}}}}}}}{|}^{2}+\vert {c}_{{d}_{0}}\cos {{{\Theta }}}_{{{{{{{{\rm{T}}}}}}}}}{|}^{2}+2|{c}_{{d}_{1}}\sin {{{\Theta }}}_{{{{{{{{\rm{T}}}}}}}}}{|}^{2}=1.0$, ${\phi }_{{d}_{+1}}^{2h\nu }={\phi }_{{d}_{-1}}^{2h\nu }$, and *φ* is limited in the range of $(\frac{1}{3}\pi,\frac{2}{3}\pi )$ and ($\frac{4}{3}\pi$, $\frac{5}{3}\pi$).

We further extend the partial-wave-resolved phase shifts reconstruction method into the case of neon and argon atoms with the help of the *m*-resolved partial-wave proportions from the theoretical simulation. The PADs coupled to *P*_0_ and *P*_±1_ states can be described by:

6 $${I}_{{{{{{{{\rm{e}}}}}}}}}^{{P}_{0}}(\theta,\varphi ) \sim	 \left | \left[{c}_{{p}_{0}}^{{P}_{0}}Y_{10}(\theta,\varphi ){e}^{-i{\phi }_{{p}_{0}}^{{P}_{0}}}+{c}_{{f}_{0}}^{{P}_{0}}Y_{30}(\theta,\varphi ){e}^{-i{\phi }_{{f}_{0}}^{{P}_{0}}}\right]\cos {{{\Theta }}}_{{{{{{{{\rm{T}}}}}}}}}\right.\\ 	+\left[-i{c}_{{p}_{1}}^{{P}_{0}}\left({Y}_{1-1}(\theta,\varphi ){e}^{-i{\phi }_{{p}_{-1}}^{{P}_{0}}}+{Y}_{11}(\theta,\varphi ){e}^{-i{\phi }_{{p}_{1}}^{{P}_{0}}}\right)\right.\\ 	 {\left.+\left.i{c}_{{f}_{1}}^{{P}_{0}}\left({Y}_{3-1}(\theta,\varphi ){e}^{-i{\phi }_{{f}_{-1}}^{{P}_{0}}}+{Y}_{31}(\theta,\varphi ){e}^{-i{\phi }_{{f}_{1}}^{{P}_{0}}}\right)\right]\sin {{{\Theta }}}_{{{{{{{{\rm{T}}}}}}}}}\right | }^{2}.$$

7 $${I}_{{{{{{{{\rm{e}}}}}}}}}^{{P}_{\pm 1}}(\theta,\varphi ) \sim	 \left | \left[{c}_{{p}_{\mp 1}}^{{P}_{\pm 1}}{Y}_{1\mp 1}(\theta,\varphi ){e}^{-i{\phi }_{{p}_{\mp 1}}^{{P}_{\pm 1}}}+{c}_{{f}_{\mp 1}}^{{P}_{\pm 1}}{Y}_{3\mp 1}(\theta,\varphi ){e}^{-i{\phi }_{{f}_{\mp 1}}^{{P}_{\pm 1}}}\right]\cos {{{\Theta }}}_{{{{{{{{\rm{T}}}}}}}}}\right.\\ 	+{\left.\left[-i{c}_{{p}_{0}}^{{P}_{\pm 1}}{Y}_{10}(\theta,\varphi ){e}^{-i{\phi }_{{p}_{0}}^{{P}_{\pm 1}}}+i{c}_{{f}_{0}}^{{P}_{\pm 1}}{Y}_{30}(\theta,\varphi ){e}^{-i{\phi }_{{f}_{0}}^{{P}_{\pm 1}}}+i{c}_{{f}_{\mp 2}}^{{P}_{\pm 1}}{Y}_{3\mp 2}(\theta,\varphi ){e}^{-i{\phi }_{{f}_{\mp 2}}^{{P}_{\pm 1}}}\right]\sin {{{\Theta }}}_{{{{{{{{\rm{T}}}}}}}}}\right | }^{2}.$$

The angle-resolved two-photon transition phase distributions are described as:

8 $${\phi }_{{P}_{0}}^{2h\nu }(\theta,\varphi ) \sim	 \arg \left\{\left[{c}_{{p}_{0}}^{{P}_{0}}{Y}_{10}(\theta,\varphi ){e}^{-i{\phi }_{{p}_{0}}^{{P}_{0}}}+{c}_{{f}_{0}}^{{P}_{0}}{Y}_{30}(\theta,\varphi ){e}^{-i{\phi }_{{f}_{0}}^{{P}_{0}}}\right]\cos {{{\Theta }}}_{{{{{{{{\rm{T}}}}}}}}}\right.\\ 	+\left[-i{c}_{{p}_{1}}^{{P}_{0}}\Big({Y}_{1-1}(\theta,\varphi ){e}^{-i{\phi }_{{p}_{-1}}^{{P}_{0}}}+{Y}_{11}(\theta,\varphi ){e}^{-i{\phi }_{{p}_{1}}^{{P}_{0}}}\Big)\right.\\ 	+\left.\left.i{c}_{{f}_{1}}^{{P}_{0}}\Big({Y}_{3-1}(\theta,\varphi ){e}^{-i{\phi }_{{f}_{-1}}^{{P}_{0}}}+{Y}_{31}(\theta,\varphi ){e}^{-i{\phi }_{{f}_{1}}^{{P}_{0}}}\Big)\right]\sin {{{\Theta }}}_{{{{{{{{\rm{T}}}}}}}}}\right\}.$$

9 $${\phi }_{{P}_{\pm 1}}^{2h\nu }(\theta,\varphi ) \sim	 \arg \left\{\left[{c}_{{p}_{\mp 1}}^{{P}_{\pm 1}}{Y}_{1\mp 1}(\theta,\varphi ){e}^{-i{\phi }_{{p}_{\mp 1}}^{{P}_{\pm 1}}}+{c}_{{f}_{\mp 1}}^{{P}_{\pm 1}}{Y}_{3\mp 1}(\theta,\varphi ){e}^{-i{\phi }_{{f}_{\mp 1}}^{{P}_{\pm 1}}}\right]\cos {{{\Theta }}}_{{{{{{{{\rm{T}}}}}}}}}\right.\\ 	+\left.\left[-i{c}_{{p}_{0}}^{{P}_{\pm 1}}{Y}_{10}(\theta,\varphi ){e}^{-i{\phi }_{{p}_{0}}^{{P}_{\pm 1}}}+i{c}_{{f}_{0}}^{{P}_{\pm 1}}{Y}_{30}(\theta,\varphi ){e}^{-i{\phi }_{{f}_{0}}^{{P}_{\pm 1}}}+i{c}_{{f}_{\mp 2}}^{{P}_{\pm 1}}{Y}_{3\mp 2}(\theta,\varphi ){e}^{-i{\phi }_{{f}_{\mp 2}}^{{P}_{\pm 1}}}\right]\sin {{{\Theta }}}_{{{{{{{{\rm{T}}}}}}}}}\right\}.$$

Thus, the experimentally measured PAD is the sum of Eq. (6) and Eq. (7) and the angle-resolved relative atomic phase shifts result from the incoherent sum of ${\phi }_{{P}_{0}}^{2h\nu }(\theta,\varphi )$ and ${\phi }_{{P}_{\pm 1}}^{2h\nu }(\theta,\varphi )$ weighted by the relative ratio of photoelectrons coupled to *P*_0_- and *P*_±1_-residual ionic states. The extracted *m*-resolved partial-wave two-photon transition phase shifts ${\phi }_{lm}^{2h\nu }$ is the proportion weighted sum of ${\phi }_{lm}^{{P}_{0}}$ and ${\phi }_{lm}^{{P}_{\pm 1}}$.

## Supplementary information

Supplementary Information

## Data Availability

The data that supports the main figures within this study is available from the Zenodo database with 10.5281/zenodo.6925094(https://zenodo.org/record/6925094#.YuKOWXZBybg).

## Code availability

The RMT theoretical simulation codes are shared in gitlab (https://gitlab.com/Uk-amor/RMT/rmt), and the other codes that support the findings of this study are available from the corresponding author upon reasonable request.
