# Supplementary material for: Atomic partial wave meter by attosecond coincidence metrology
Source: Nat Commun. 2022 Aug 29;13:5072. doi: 10.1038/s41467-022-32753-8 (PMC9424306; doi:10.1038/s41467-022-32753-8)
Supplement: Supplementary file 1 — Supplementary Information [file 41467_2022_32753_MOESM1_ESM.pdf]

Supplementary Information for
Atomic partial wave meter by attosecond coincidence metrology

Wenyu Jiang[1], Gregory S. J. Armstrong[2], Jihong Tong[1], Yidan Xu[1], Zitan Zuo[1], Junjie Qiang[1], Peifen Lu[1], Daniel D. A. Clarke[3], Jakub Benda[4], Avner Fleischer[5], Hongcheng Ni[1,6], Kiyoshi Ueda[1], Hugo W. van der Hart[2], Andrew C. Brown[2,†], Xiaochun Gong[1,6,†], Jian Wu[1,6,7,†]

[1]*State Key Laboratory of Precision Spectroscopy, East China Normal University, Shanghai, China*
[2]*Centre for Theoretical Atomic, Molecular and Optical Physics, School of Mathematics and Physics, Queen's University Belfast. University Road, Belfast, BT7 1NN, Northern Ireland, UK*
[3]*School of Physics and CRANN Institute, Trinity College Dublin, Dublin 2, Ireland*
[4]*Institute of Theoretical Physics, Faculty of Mathematics and Physics, Charles University, V Holešovičkách 2, 180 00 Prague 8, Czech Republic*
[5]*Raymond and Beverly Sackler Faculty of Exact Science, School of Chemistry and Center for Light-Matter Interaction, Tel Aviv University, Tel-Aviv 6997801, Israel*
[6]*Collaborative Innovation Center of Extreme Optics, Shanxi University, Taiyuan, Shanxi 030006, China*
[7]*CAS Center for Excellence in Ultra-intense Laser Science, Shanghai 201800, China*
† *e-mail: andrew.brown@qub.ac.uk, xcgong@lps.ecnu.edu.cn, jwu@phy.ecnu.edu.cn*

Contents

Supplementary Note 1: XUV-APT and attosecond photoelectron spectra

Supplementary Note 2: Photoionization time delay reconstruction routine

Supplementary Note 3: Theoretical simulation

3.1 Full quantum simulation: RMT

3.2 Full partial wave analysis

3.3 Intensity dependence of the two-photon ionization process in helium, neon and argon

3.4 Analytical two photon ionization model: soft photon approximation

Supplementary Note 1: XUV-APT and attosecond photoelectron spectra

Supplementary Figures 1 (a) and (b) show the measured RABBITT trace integrated over the photoemission angles in the argon atom, and the reconstructed spectrum via FROG-CRAB through the algorithm of PCGPA[1], respectively. The photoelectron kinetic energy in our measurements was calibrated by the ionization potential of the $3p$ electron of argon atom, I_{p} = 15.76 eV. As shown in Supplementary Fig. 1(c), the pulse duration of the XUV-APT is estimated to be 320 attoseconds, the relative chirp distribution of the XUV-APT is calibrated through the effective photoionization time delay of argon, where the chirp of the H19 is set to be zero.

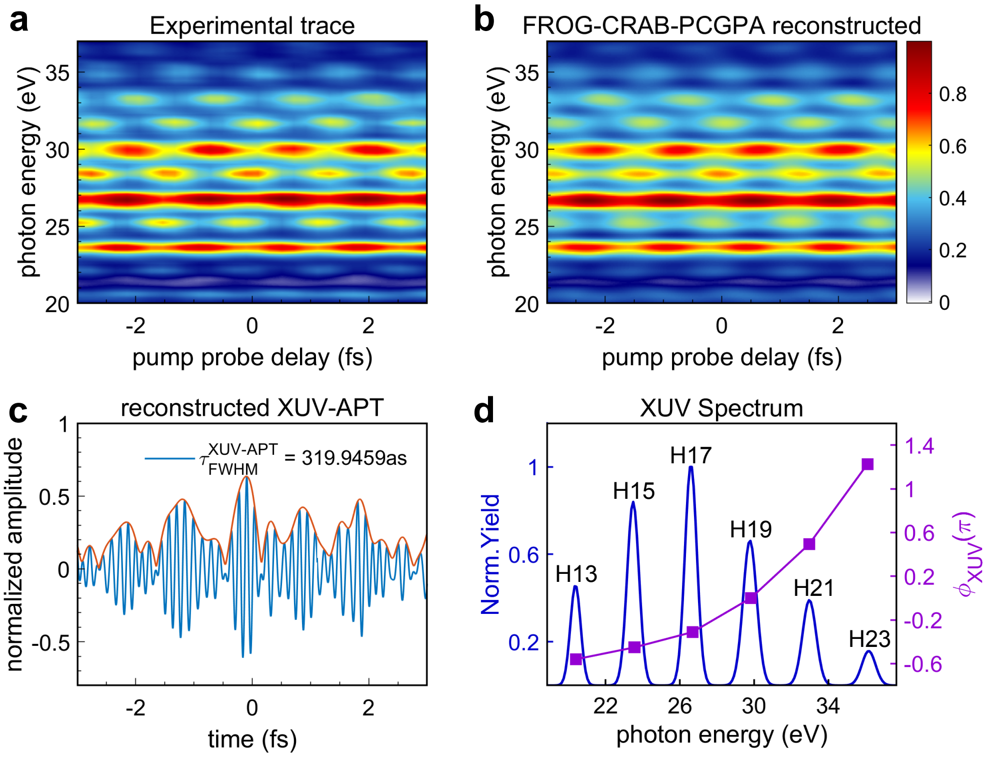

Supplementary Fig. 1: XUV-APT characterization. (**a**) Experimentally measured RABBITT spectrum in argon atoms at $\Theta_{\mathrm{T}} = 0°$. The photoelectron yield is normalized to its peak value. (**b**) Reconstructed RABBITT spectrum. (**c**) Reconstructed electric field of the XUV-APT. (**d**) The spectrum of XUV-APT. The blue line shows the measured photoelectron energy spectrum of argon shifted by the ionization potential (15.76eV) and the purple dots display the harmonic phase, ϕ_{XUV} relative to the 19th harmonic.

Supplementary Figures 2(a-h) show the experimentally measured and theoretically calcu-

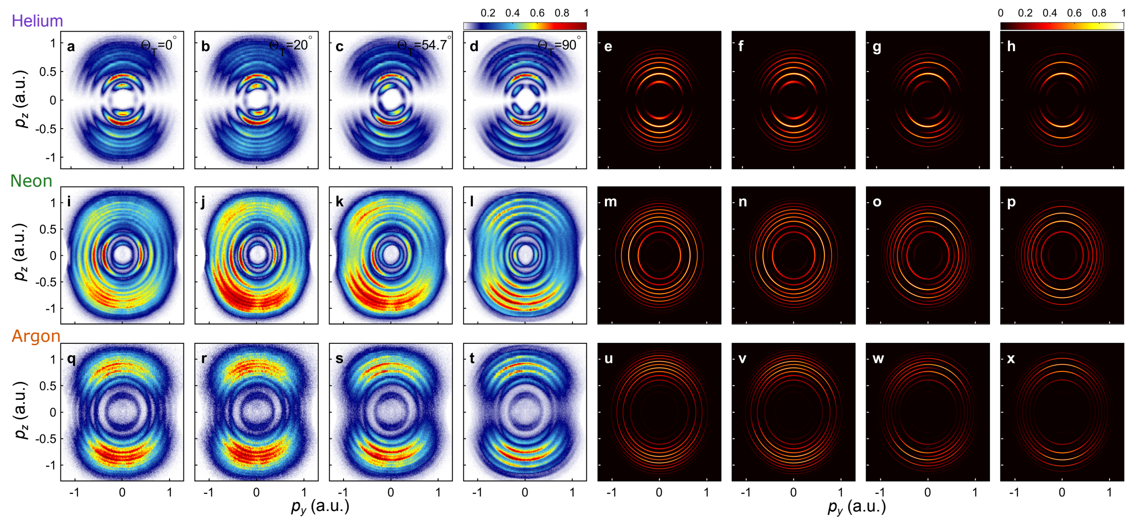

Supplementary Fig. 2: Photoelectron angular distributions. (**a-d**) Experimentally measured and (**e-h**) theoretically calculated PADs of helium averaged over the pump-probe delay, under the skewed angle of $\Theta_{\mathrm{T}} =$ (**a**) $0°$, (**b**) $20°$, (**c**) $54.7°$, (**d**) $90°$. (**i-p**) and (**q-x**) as in (**a-h**) but for neon and argon atom results, respectively.

lated photoelectron angular distributions (PADs) from helium, where the polarization of the NIR pulse rotates from a parallel ($\Theta_{\mathrm{T}} = 0°$) to a perpendicular ($\Theta_{\mathrm{T}} = 90°$) orientation with respect to the polarization axis of the XUV-APT. At $\Theta_{\mathrm{T}} = 0°$, the PAD of the main peaks is aligned parallel to the polarization axis of XUV-APT, whose asymmetry parameter is larger than zero. However, the PAD of the sidebands shows a four-fold distribution analogous to the wave character of d_0 wave. The left-right asymmetry of the photoelectron yield is not from the physical interaction but the inhomogeneous detection efficiency of the MCP detectors. Since it does not affect the reconstruction of the oscillation phase of the sidebands, we keep the raw results without any additional symmetric operations. As the skew angle Θ_{T} increases from $0°$ to $54.7°$, the PAD of the sidebands follows the rotation of the skewed polarization direction of the NIR pulse, which breaks the plane mirror symmetry along the z-axis. When the skew angle Θ_{T} reaches $90°$, both the plane mirror symmetry and reflection symmetry reappear along the polarization direction of the XUV-APT and NIR pulse. A fully symmetric four-fold structure is observed in PADs of sidebands, and it directly shows the angular character of the $d_{\pm 1}$ wave following the dipole transition rules. This kind of four-fold symmetry is also visible in the low kinetic energy range for argon, due to the increment of the proportion of the f-waves.

Supplementary Figures 2(i-p) show the PADs of neon for the same set of skew angles as in

Supplementary Figs. 2 (a-h). The main bands of neon, with photoelectron kinetic energy below 6 eV, are aligned perpendicular to the polarization direction of the XUV-APT with negative asymmetry parameters, due to the relative ratio between the final s and d-partial waves[2–4]. However, the photoelectron PAD of the sidebands keeps following the polarization axis of the NIR pulse as Θ_{T} varies from $0°$ to $90°$ [5,6]. Supplementary Figures 2(q-x) show the PADs of argon in the same conditions. The asymmetry parameters of the main bands of argon are larger than zero with the PADs aligning along the XUV-APT polarization axis.

Supplementary Figures 3(a-h), (i-p), and (q-x) display the attosecond photoelectron spectra as a function of the pump-probe time delay for helium, neon and argon under the skew angle of $\Theta_{\mathrm{T}} = 0°, 20°, 54.7°, 90°$, respectively. Differently from argon, the lowest order sidebands in helium (SB16) and neon (SB14) originate from interference between the continuum electron and high excited Rydberg states[7].

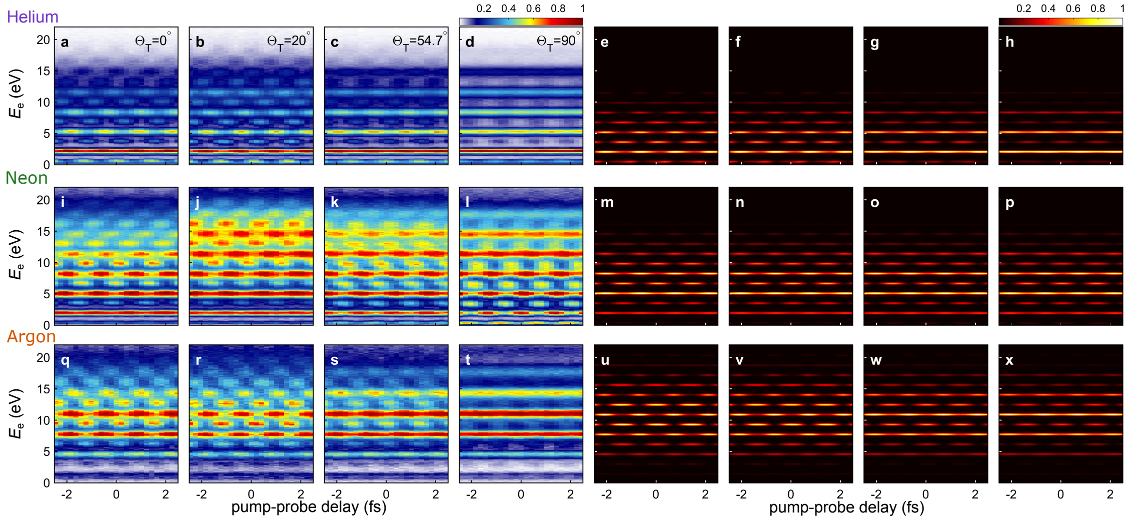

Supplementary Fig. 3: Attosecond photoelectron energy spectra. (**a-d**) Experimentally measured and (**e-h**) theoretically calculated photoelectron energy spectra of helium as a function of the pump-probe delay under the skewed angle of $\Theta_{\mathrm{T}} =$ (**a**) $0°$, (**b**) $20°$, (**c**) $54.7°$, (**d**) $90°$. (**i-p**) and (**q-x**) as same as (**a-d**) but for neon and argon results, respectively.

Supplementary Note 2: Photoionization time delay reconstruction routine

The sideband amplitude shows an oscillation as a function of the relative pump-probe time delay between the XUV-APT and the NIR pulse. As illustrated in the main text, the initial phases

of the sidebands encode the dispersion of the XUV-APT and the scattering phase shifts during the photoionization process[8]. Supplementary Figure 4 shows the experimentally measured angle-integrated attosecond photoelectron spectra of the helium atom at a skew angle Θ_{T} = $0°$. To acquire the oscillation phase and amplitude, we perform a fast Fourier transform (FFT) analysis of the RABBITT spectrum. The oscillation term around $2\omega_{\mathrm{NIR}}$ can be fitted in the time domain as $S(\tau, \theta) = a_0 \cos(2\omega_{\mathrm{NIR}}\tau + \phi_0(\theta)) + b_0$, where τ is the pump-probe time delay and b_0 is the averaged yield of a given sideband, a_0 and ϕ_0 are the oscillation amplitude and initial phase shifts. The initial phase ϕ_0 of the sideband can be decomposed into the phase difference between consecutive harmonics, $\phi_{\mathrm{XUV-APT}}$, and the intrinsic atomic phase shifts $\phi^{2h\nu}(\theta)$ during the photoionization process[9–13], $\phi_0(\theta) = \phi_{\mathrm{XUV-APT}} + \phi^{2h\nu}(\theta)$.

Supplementary Note 3: Theoretical simulation

3.1 Full quantum simulation: RMT

RMT provides a non-perturbative, *ab initio* solution of the time-dependent Schrödinger equation (TDSE) for multielectron atoms and molecules. Theoretical[14,15] and computational[16] details have been reported in full elsewhere, so here we provide only a brief overview.

To describe an atomic system containing $N+1$ electrons we divide the physical space around the nucleus into two regions. The inner region, confined to small radial distances from the nucleus, requires a full account of all electronic interactions including electron exchange which is accomplished using a configuration-interaction approach. A single electron may escape this inner region, at which point it is spatially isolated from the residual N-electron core, and electron exchange may be neglected. In the outer region, then, a single, ionized electron moves under the influence of both the long-range potential of the N-electron ion and the laser field. The full complexity of the many-body system is thus confined to the small, inner region, while the outer region is effectively a single-active electron problem. This division of space is necessary to reduce the computational load of describing the electron exchange in particular, especially for ionisation problems where the electron may travel far from the nucleus.

RMT leverages this division of space further by employing a markedly different numerical scheme in each region. In the inner region, a B-spline basis set is used to ensure accurate and efficient determination of the multielectron wavefunction. By contrast, in the outer region a

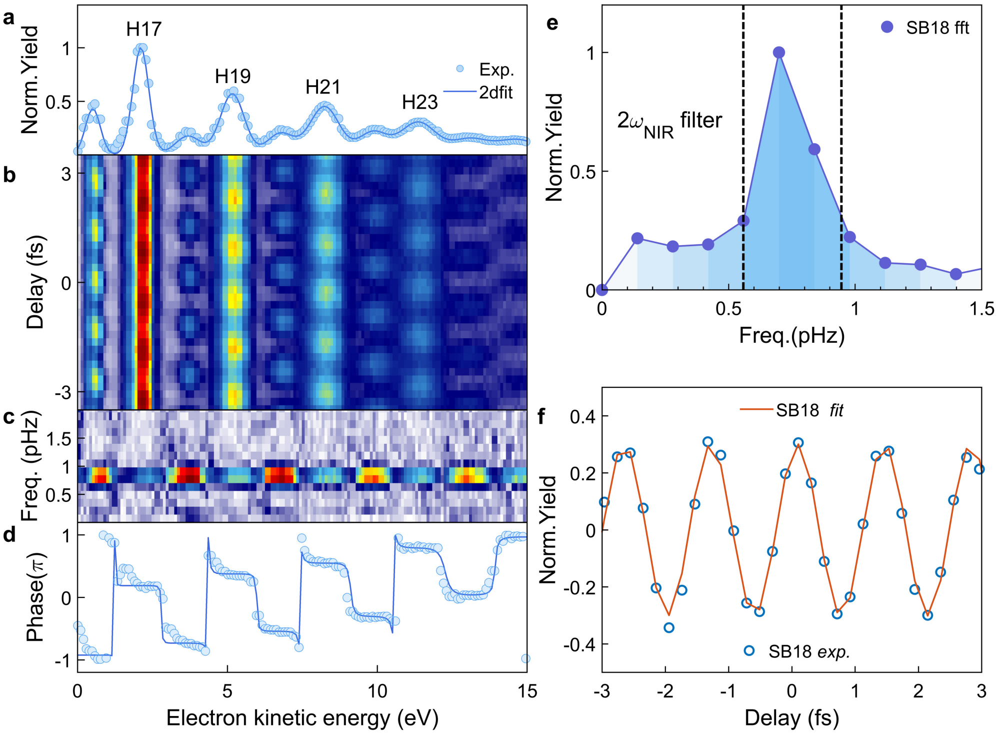

Supplementary Fig. 4: Sideband phase reconstruction. (**a**) Photoelectron kinetic energy spectrum of helium. (**b**) Photoemission angle-integrated RABBITT trace under the skew angle of $\Theta_{\mathrm{T}} = 0°$. (**c**) The oscillation amplitude of the RABBITT trace. (**d**) The sideband phase distribution around the oscillation frequency of $2\omega_{\mathrm{NIR}}$. The blue dots and solid line show the measured and fitted results. (**e**) Oscillation amplitudes for SB18 with a kinetic energy window of 0.8eV. The two dashed lines indicate the filter window. (**f**) Normalized oscillation trace of SB18. The blue dots and solid lines represent the measured and fitted results.

grid-based finite difference scheme is employed. This also facilitates the use of a sophisticated, multi-layered parallelisation scheme, allowing RMT to be deployed on massively-parallel high-performance computers.

The $N+1$-electron atom is constructed by coupling a continuum electron to the N-electron ionic states. This additional electron may occupy a bound orbital, yielding the ground and excited states, or it may be in the continuum, offering an accurate description of the ionized, N-electron ion and its interaction with the outgoing electron. This also allows the description of the outgoing wavepacket to be cast in terms of ionization ‘channels’, with each channel representing the emis-

sion of an electron with a particular set of quantum numbers, coupled to a specific ionic state. This channel formalism is key in the present work, as the outgoing electron may be decoupled from the residual in order to provide the partial wave decomposition of the continuum wavefunction.

For both argon and neon, the atomic structure description is based on the R-matrix basis reported elsewhere[17], and includes all outgoing electron emission channels up to maximum total angular momentum of $L_{\max} = 3$ (convergence of the results was checked with $L_{\max} = 5$). The total number of $LM_LS\pi$ symmetries included in the calculation is 31. The inner region radius is set at 20 a_0, where a_0 is the Bohr radius. For helium this inner region radius is 15 a_0, and the atomic structure description is the so-called '1T' description reported elsewhere[18]. This includes electron emission channels up to $L_{\max} = 5$ attached to the ${}^2S^e$ ionisation threshold, and a single $1s$ orbital in the description of He^+. This gives a total of 36 $LM_LS\pi$ symmetries. For all calculations, the outer region is 5160 a_0, which is sufficiently large to ensure it is never reached by the outgoing wavepacket, which can cause non-physical reflections that obscure the resulting wavefunction. The wavefunction for the outgoing electron is obtained by decoupling it from the wavefunction for the residual ion[19]. It is subsequently transformed into momentum space through a Fourier Transform.

The laser parameters are reported in the main text, and are chosen as a compromise between the experimental values, and those which will enable numerically stable and tractable calculations. The electric field profiles of the NIR and XUV-APT pulses are shown in Supplementary Fig. 5, along with the frequency comb of the XUV-APT.

3.2 Full partial wave analysis

The partial wave transition pathways for two-photon ionization of helium are illustrated in Supplementary Table 1. Supplementary Tables 2 and 3 show the partial wave transition pathways of the neon and argon atoms. The final states in neon and argon involve several, degenerate ionic states. The final state of the coupled (ion + photoelectron) system is required to decompose the partial wave channels properly. The selection rules for the various transitions enforce that the total magnetic quantum number must not change ($\Delta m = 0$) when $\Theta_{\mathrm{T}} = 0°$ and that $\Delta m = \pm 1$ for $\Theta_{\mathrm{T}} = 90°$. Only for $\Theta_{\mathrm{T}} = 0°$ and $90°$, can the final, total symmetry be determined exactly, and the resulting PADs can then be understood as arising from specific m-values of the outgoing electron. For the other skew angles, the outgoing electrons with initial magnetic quantum number $m_{\mathrm{i}} = 0$ can transition to a continuum state with final magnetic quantum number $m_{\mathrm{f}} = 0$ or $m_{\mathrm{f}} = \pm 1$. In

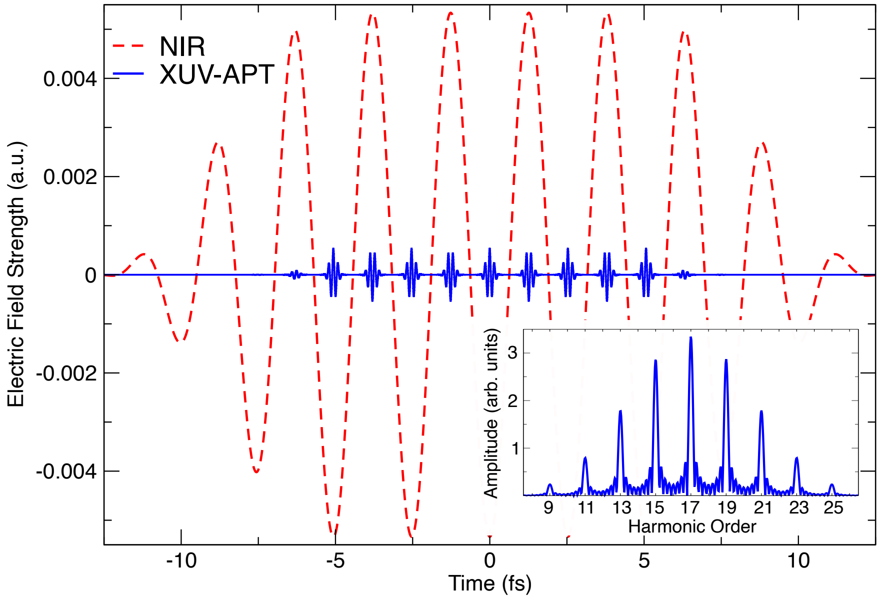

Supplementary Fig. 5: The pulse profiles employed in the RMT calculations. The NIR pulse is shown in the dashed, red line, and the XUV-APT in the solid, blue line. The inset shows the Fourier Transform of the XUV-APT, which models the frequency comb of the experimental pulse-train.

this case, one needs to consider the output of a full quantum calculation, and to uncouple, using the appropriate Clebsch-Gordan coefficients[20], the outer electron from the residual ion.

Supplementary Fig. 6 shows the PADs of each partial wave involved in the two-photon ionization of the helium, neon and argon. The PAD of the s-wave is isotropic. The PADs of the partial waves p_0 and $p_{\pm 1}$ present a perpendicular polarization, centred along z and y axis, respectively. As compared to the partial wave of p_0, the PAD of d_0 wave is given by the squared spherical harmonic $|Y_{20}(\theta, \phi = 0)|^2 \sim (3\cos^2\theta - 1)^2$, which has a four-fold symmetry with maxima at $\theta = 0°, \pm 90°, 180°$. The four-fold angular structure of $d_{\pm 1}$ results from the $|Y_{2\pm 1}(\theta, \phi = 0)|^2 \sim (\sin\theta^2 \cos\theta^2)$, which has zero nodes at $\theta = 0°, \pm 90°$ and $180°$, and maxima at $\theta = \pm 45°, \pm 135°$. The PADs of the partial wave of f_0 displays an angular character of $|Y_{30}(\theta, \phi = 0)|^2 \sim (5\cos^3\theta - 3\cos\theta)^2$ with a peak position at $\theta = \pm 39.2°, \pm 90°$ and $\pm 140.8°$. The PADs for $f_{\pm 1}$ electrons displays a six-fold symmetry given by $|Y_{3\pm 1}(\theta, \phi = 0)|^2 \sim (5\cos^2\theta - 1)^2 \sin^2\theta$, with

Θ_T	Δm	$(M_i^{ion}, m_i^{elec.})$	$lm_i^{elec.}$	$(M_f^{ion}, m_f^{elec.})$	$lm_f^{elec.}$
0°	0	$(0, 0)$	s	$(0, 0)$	d_0, s
90°	±1	$(0, 0)$	s	$(0, \pm1)$	$d_{\pm1}$

Supplementary Table 1: The atomic symmetry character of helium in two-photon transition. The quantum states of ion and photoelectron in two-photon ionization in helium at $\Theta_T = 0°$ and 90°. $M_{i/f}^{ion}$ indicate the initial and final M value of the singly-charged residual ion, $m_{i/f}^{elec.}$ indicate the initial and final m value of the ejected electron.

Δm	$(M_i^{ion}, m_i^{elec.})$	$lm_i^{elec.}$	$(M_f^{ion}, m_f^{elec.})$	$lm_f^{elec.}$
0	$(0, 0)$	p_0	$(0, 0)$	p_0, f_0
0	$(\mp1, \pm1)$	$p_{\pm1}$	$(\mp1, \pm1)$	$p_{\pm1}, f_{\pm1}$

Supplementary Table 2: The atomic symmetry character of neon and argon in two-photon transition. The initial and final quantum states of ion and photoelectron in neon/argon at $\Theta_T = 0°$. $M_{i/f}^{ion}$ indicate the initial and final M value of the singly-charged residual ion, $m_{i/f}^{elec.}$ indicate the initial and final m value of the ejected electron.

Δm	$(M_i^{ion}, m_i^{elec.})$	$lm_i^{elec.}$	$(M_f^{ion}, m_f^{elec.})$	$lm_f^{elec.}$
±1	$(0, 0)$	p_0	$(0, \pm1)$	$p_{\pm1}, f_{\pm1}$
±1	$(\mp1, \pm1)$	$p_{\pm1}$	$(\mp1, 0)$	p_0, f_0
±1	$(\mp1, \pm1)$	$p_{\pm1}$	$(\mp1, \pm2)$	$f_{\pm2}$

Supplementary Table 3: The atomic symmetry character of neon and argon in two-photon transition. The quantum states of ion and photoelectron in neon/argon at $\Theta_T = 90°$. $M_{i/f}^{ion}$ indicate the initial and final M value of the singly-charged residual ion, $m_{i/f}^{elec.}$ indicate the initial and final m value of the ejected electron.

a peak positions at $\theta = 0°, \pm 63.5°, \pm 116.5°$ and $180°$. The angular distribution of $f_{\pm 2}$ electrons is given by $|Y_{3\pm 2}(\theta, \phi = 0)|^2 \sim \cos^2\theta \sin^4\theta$ with four maxima at $\theta = \pm 54.7°, \pm 125.3°$ and a mirror reflection symmetry along $\theta = 0°$ or $180°$.

As shown in Supplementary Fig. 7, the PADs of the s-wave in helium maintain an isotropic distribution across all skew angles, while the d-wave follows the rotation of the NIR polarization field, and reaches pure $d_{\pm 1}$ partial waves at $\Theta_{\mathrm{T}} = 90°$. The relative weights and phase shifts of each partial wave in neon and argon are sensitive to the exact potential of the atomic system. Supplementary Figures 8 (a-e) present the PADs of p-waves with the residual-ion state of P_0 symmetry for neon at $\Theta_{\mathrm{T}} = 0°, 20°, 54.7°, 75°$ and $90°$, and the Supplementary Figs. 8 (f-j) shows the PADs of f-waves for the same skew angles. As the skew angle increases from $20°$ to $75°$, the interference between the $p_{\pm 1}$- and p_0-waves results in the rotated PADs and plane mirror symmetry breaking along z-axis. When $\Theta_{\mathrm{T}} = 90°$, the plane mirror symmetry appears again. As compared to the p-wave, the six-fold PADs of the f-wave shows a similar rotation dependence as a function of Θ_{T}.

Supplementary Figures 9 (a-e, f-j) present the PADs of p and f electrons with the residual-ion states of $P_{\pm 1}$ symmetry of neon under the same skew angles as in Supplementary Fig. 8. Here, the alignment of the PADs of p-waves rotates from the y-axis to the z-axis as Θ_{T} changes from $0°$ to $90°$. Supplementary Figures 8(k-t) and Supplementary Figs. 9(k-t) display the l-resolved PADs of argon as in Supplementary Figs. 8(a-j) and Supplementary Figs. 9 (a-j). The ionic state resolved PADs of p- and f-waves of argon have the same angular characteristics as those in neon.

Supplementary Figures 10 (a-e, f-j) show the PADs of p- and f-wave of neon including an incoherent sum over the residual-ion states with P_0 and $P_{\pm 1}$ symmetry. The PADs of p waves display an isotropic distribution at $\Theta_{\mathrm{T}} = 0°$, but a more polarized character at other skew angles. The PADs of f waves display a four-fold symmetry over all skew angles, with most of the photoelectron yield aligned along the NIR polarization axis except at $\Theta_{\mathrm{T}} = 90°$, where the f-wave PADs show a uniform four-fold structure. The PADs of the p-wave of argon in Supplementary Fig. 10 (k) at $\Theta_{\mathrm{T}} = 0°$ show the polarization character along the z-axis, which is different to neon. As shown in Supplementary Figs. 10 (p-t), the f-waves of argon display the same angular structures as those of neon.

3.3 Intensity dependence of the photoionization time delay in helium, neon and argon

To determine the effect of NIR intensity on the m-resolved partial wave phase shifts as a function of Θ_{T}, we perform RMT calculations at three different NIR intensities: $1.0\mathrm{TW/cm^2}, 0.5\mathrm{TW/cm^2}$ and $0.1\mathrm{TW/cm^2}$. Supplementary Figures 11(a-c) show the effective atomic phase shift as a function of the skew angle and intensity of the NIR field for the helium atom within the kinetic energy range from $3.7\mathrm{eV}$ to $9.9\mathrm{eV}$. As the skew angle weighted purple lines shown in Supplementary Fig. 11(a), the effective phase shift of SB18, $\phi^{2h\nu}_{\mathrm{SB18}}$, varies from -0.099π to -0.107π at 1.0TW/cm^2, whereas the $\phi^{2h\nu}_{\mathrm{SB18}}$ maintains a constant value of -0.104π over all skew angles at the NIR intensity $I_{\mathrm{NIR}} = 0.1$TW/cm^2, close to the perturbative limit.

Supplementary Figures 11(d-f) show the m-resolved partial wave phase shifts of helium as a function of Θ_{T} with the same NIR intensities as in Supplementary Figs. 11 (a-c). In the case of the NIR intensity of 1.0TW/cm^2, the phase shifts of d_0, $\phi^{2h\nu}_{d_0}$, slightly increase from -0.129π to -0.102π as the skew angle changes from 0° to $75°$. The $\phi^{2h\nu}_{d_{\pm1}}$ shows a larger increment than $\phi^{2h\nu}_{d_0}$ that ranges from -0.149π at $\Theta_{\mathrm{T}} = 20°$ to -0.109π at $\Theta_{\mathrm{T}} = 90°$. For the s-wave, the phase shift, $\phi^{2h\nu}_{s}$ is independent of Θ_{T} and keeps the same value of -0.0884π. As the NIR intensity decreases from 1.0TW/cm^2, the skew angle induced phase shift increments of d_0- and $d_{\pm1}$-waves become less obvious and the RMT simulation shows a constant lm-resolved partial wave phase shift at $0.1\mathrm{TW/cm^2}$, which for $\phi^{2h\nu}_{s}$, $\phi^{2h\nu}_{d_0}$ and $\phi^{2h\nu}_{d_{\pm1}}$ is -0.109π, -0.104π, and -0.104π, respectively.

Supplementary Figures 13(a-c) show the effective atomic phase shifts of neon with the same NIR intensities as in helium. As Θ_{T} increases from $0°$ to $90°$, the effective atomic phase shift of SB18, $\phi^{2h\nu}_{\mathrm{SB18}}$, changes from -0.0720π to -0.0367π with a maximum phase shift difference of around 0.035π. This skew angle assisted phase shift difference decreases to 0.028π and 0.023π at the NIR intensity of $I_{\mathrm{NIR}} = 0.5\mathrm{TW/cm^2}$ and $0.1\mathrm{TW/cm^2}$, respectively.

During the XUV-NIR two-photon ionization process of neon and argon, there are three possible residual-ion states, P_0 and $P_{\pm1}$, whose contributions must be summed incoherently in the final sidebands. As shown in Supplementary Fig. 14(a), each m-resolved partial wave maintains a constant phase shift over all skew angles at the perturbative limit ($I_{\mathrm{NIR}} = 0.1$ TW/cm^2). In the case of P_0, $\phi^{2h\nu}_{p_0} = -0.235\pi$, $\phi^{2h\nu}_{p_{\pm1}} = -0.038\pi$, $\phi^{2h\nu}_{f_0} = -0.0457\pi$, and $\phi^{2h\nu}_{f_{\pm1}} = -0.0459\pi$. In the case of $P_{\pm1}$, $\phi^{2h\nu}_{p_0} = -0.0465\pi$, $\phi^{2h\nu}_{p_{\pm1}} = -0.0488\pi$, $\phi^{2h\nu}_{f_0} = -0.0448\pi$, $\phi^{2h\nu}_{f_{\pm1}} = -0.0457\pi$, and $\phi^{2h\nu}_{f_{\pm2}} = -0.0471\pi$.

We find that both $p_{\pm1}$ and $f_{0/\pm1/\pm2}$ partial waves present an almost identical phase shift between different residual-ion states. The major difference appears in the p_0 partial wave. We can conclude that the skew-angle-dependent p_0 partial wave phase shift variation in Supplementary Fig. 14(c) arises from the incoherent sum over different ionic states. Since the two-photon phase shifts of $p_{\pm1}$- and $f_{0,\pm1,\pm2}$-waves only have subtle ionic-state-induced phase shift differences, the effective phase shift variation as a function of the skew angle is mainly from $\phi_{p_0}^{2h\nu}$ that changes from $\phi_{p_0}^{2h\nu}$ coupled to P_0 ionic state to $\phi_{p_0}^{2h\nu}$ coupled to $P_{\pm1}$ ionic states as the NIR field rotates from 0° to 90°.

As the NIR intensity increases from $I_{\mathrm{NIR}} = 0.1$ TW/cm^2 to $I_{\mathrm{NIR}} = 1.0$ TW/cm^2, all m-resolved partial waves show a slight variation as a function of the skew angle, and p_0 generally shows a stronger skew angle dependence than $p_{\pm1}$ and $f_{0,\pm1,\pm2}$.

Supplementary Figures 14 (j-r) shows the same results but in argon, and its skew angle dependence and NIR intensity dependence is analogous to neon. However, as shown in Supplementary Figs. 14 (j-l), the partial wave phase shift in the perturbative limit is $-0.119\pi, 0.0537\pi, -0.0140\pi$ and -0.0145π for $p_0, p_{\pm1}, f_0$ and $f_{\pm1}$-waves coupled to the P_0 residual-ion state, and -0.0172π, -0.0188π, -0.0136π, -0.0140π and -0.0137π for $p_0, p_{\pm1}, f_0, f_{\pm1}$ and $f_{\pm2}$-waves coupled to the $P_{\pm1}$ residual-ion state. We find that the phase shifts of the p_0- and $p_{\pm1}$-waves are about 0.116π and 0.092π larger than those in neon in the case of P_0 residual-ion state, which can be mainly attributed to the short-range phase shift difference between $\delta_{2p\to s}^{\mathrm{Ne}}$ and $\delta_{3p\to s}^{\mathrm{Ar}}$ during the one-photon ionization process[23]. Supplementary Figure 15 displays the relative two-photon transition phase shifts between p- and f-waves as a function of the skew angle. In comparison with Fig. 5 in main text, the $\Delta\phi_{p_0-f_0}^{2h\nu}$ and $\Delta\phi_{p_{\pm1}-f_{\pm1}}^{2h\nu}$ phases shown here are reconstructed from the experimental results by applying the partial wave proportions predicted by the SPA model.

Supplementary Figure 16, Supplementary Fig. 17, and Supplementary Fig. 18 present the photoemission angle-resolved phase shift distributions as a function of the skew angle and NIR intensity for helium, neon, and argon atoms. The observed phase shift angular distribution is from the partial wave interference, where an incoherent sum should also be considered when the initial states are degenerate.

3.4 Analytical two photon ionization model

To extract the analytical dependence on relative polarization angle, we use the 'soft photon approximation' [24], which expresses the S-matrix element for the exchange of n NIR photons with a single XUV photon as

$$S^{(n)} = -2\pi i J_{-n}(\boldsymbol{\alpha}_{\mathrm{NIR}} \cdot \mathbf{k}_n) \exp(-i\phi_{\mathrm{XUV}} + n\phi_{\mathrm{NIR}}) \left\langle \chi_{\mathbf{k}_n} | \boldsymbol{\epsilon}_{\mathrm{XUV}} \cdot \mathbf{p} | \Psi_{\mathrm{i}} \right\rangle . \tag{S1}$$

Here, $\boldsymbol{\alpha}_{\mathrm{NIR}}$ and $\boldsymbol{\epsilon}_{\mathrm{XUV}}$ are the polarization vectors of the NIR and XUV fields respectively, $\mathbf{k}_n$ is the sideband momentum, ϕ_{NIR} and ϕ_{XUV} are their respective carrier-envelope phases, Ψ_{i} is the initial-state wave function, and $\chi_{\mathbf{k}_n}$ is the final-state wave function.

Typically, the final state is expressed as a plane wave, but the Coulomb potential may be taken into account using the appropriate expansion

$$\chi_{\mathbf{k}_n} = \frac{4\pi}{kr} \sum_{l,m} i^l e^{i\sigma_l} \mathcal{F}_l(\eta, kr) Y^*_{l,m}(\theta_k, \phi_k) Y_{l,m}(\theta_r, \phi_r), \tag{S2}$$

where $\sigma_l = \arg\left[\Gamma\left(l + 1 + i\eta\right)\right]$ is the Coulomb phase shift, and $\mathcal{F}_l(\eta, kr)$ is the regular Coulomb function, with $\eta = -Z/k$. We align the XUV field at a relative angle Θ_{T} to the z-axis, and assume the lowest order pathways, which for the RABBITT processes of interest involve the $n = 1$ (absorption) and $n = -1$ (emission) contributions. The angle dependence is obtained by expanding the $n = \pm 1$ Bessel functions for small arguments:

$$J_{\pm 1}(x) \sim x, \tag{S3}$$

so that for an arbitrary relative polarization

$$J_{\pm 1}(\boldsymbol{\alpha}_{\mathrm{NIR}} \cdot \mathbf{k}_1) \sim k_1 \left[\cos\Theta_{\mathrm{T}} \cos\theta_k + \sin\Theta_{\mathrm{T}} \sin\theta_k \sin\phi_k\right] . \tag{S4}$$

So, the amplitudes become

$$S^{(n=\pm1)} \sim -2\pi i e^{-i(\phi_{2q\mp1}\pm\phi_L)} \left[\cos\Theta_{\mathrm{T}}\cos\theta_k + \sin\Theta_{\mathrm{T}}\sin\theta_k\sin\phi_k\right] \langle \chi_{\mathbf{k}_n}|p_z|\psi_{l_{\mathrm{i}}m_{\mathrm{i}}}\rangle , \tag{S5}$$

where $\phi_{2q\pm1}$ are the carrier-envelope phases of harmonics $2q \pm 1$, and $\psi_{l_{\mathrm{i}}m_{\mathrm{i}}}$ is the initial-state wavefunction with orbital angular momentum l_{i} and magnetic quantum number m_{i}.

helium For helium, the angular integral is given by

$$\langle Y_{lm}|\cos\theta_r|Y_{00}\rangle = \delta_{l1}\delta_{m0}\,\frac{1}{\sqrt{3}}, \tag{S6}$$

and so only the p_0 intermediate state contributes, so that Eq. (S14) becomes

$$I_z = Y_{10}(\theta_k,\phi_k)e^{-i(\pi/2+\sigma_1)}\frac{R_{ps}}{\sqrt{3}}, \tag{S7}$$

where

$$R_{ps} = \int kr\mathcal{F}_1(\eta,kr)\left[\frac{d}{dr} - \frac{1}{r}\right] f_{00}(r)dr. \tag{S8}$$

Using Eq. (S7), the amplitudes in Eq. (S5) are given by

$$S^{(n=\pm1)} \sim -2\pi i e^{-i(\phi_{2q\mp1}\pm\phi_L+\pi/2+\sigma_1)} \left[\cos\Theta_{\mathrm{T}}\cos\theta_k + \sin\Theta_{\mathrm{T}}\sin\theta_k\sin\phi_k\right] Y_{10}(\theta_k,\phi_k)\frac{R_{ps}}{\sqrt{3}}. \tag{S9}$$

Expressing $\cos\theta_k$ and $\sin\theta_k\sin\phi_k$ as spherical harmonics, and then contracting the resulting products of spherical harmonics, Eq. (S9) may be written as

$$
\begin{aligned}
S^{(\pm 1)} \sim & -2\pi i e^{-i(\phi_{2q\mp 1} \pm \phi_L + \pi/2 + \sigma_1)} \frac{R_{ps}}{\sqrt{3}} \cos\Theta_{\mathrm{T}} \left[\frac{1}{\sqrt{3}} Y_{00}(\theta_k, \phi_k) + \frac{2}{\sqrt{15}} Y_{20}(\theta_k, \phi_k) \right] \\
& -2\pi i e^{-i(\phi_{2q\mp 1} \pm \phi_L + \pi/2 + \sigma_1)} \frac{R_{ps}}{\sqrt{3}} \sin\Theta_{\mathrm{T}} i \sqrt{\frac{1}{10}} \left[Y_{21}(\theta_k, \phi_k) + Y_{2-1}(\theta_k, \phi_k) \right], \qquad \text{(S10)} \\
\sim & -2\pi i e^{-i(\phi_{2q\mp 1} \pm \phi_L + \pi/2 + \sigma_1)} R_{ps} \cos\Theta_{\mathrm{T}} \left[\frac{1}{3} Y_{00}(\theta_k, \phi_k) + \frac{2}{\sqrt{45}} Y_{20}(\theta_k, \phi_k) \right] \\
& -2\pi i e^{-i(\phi_{2q\mp 1} \pm \phi_L + \pi/2 + \sigma_1)} R_{ps} \sin\Theta_{\mathrm{T}} i \sqrt{\frac{1}{30}} \left[Y_{21}(\theta_k, \phi_k) + Y_{2-1}(\theta_k, \phi_k) \right]. \qquad \text{(S11)}
\end{aligned}
$$

The photoelectron yield for each partial wave lm is then calculated by squaring the coefficient of the spherical harmonic Y_{lm}. From Eq. (S11), the yields of s and d_0 electrons depend only on $\cos^2\Theta_{\mathrm{T}}$ as

$$
\mathcal{P}_s \sim \frac{|R_{ps}|^2}{9} \cos^2\Theta_{\mathrm{T}}; \quad \mathcal{P}_{d_0} \sim \frac{4|R_{ps}|^2}{45} \cos^2\Theta_{\mathrm{T}}, \qquad \text{(S12)}
$$

and the yield of $d_{\pm 1}$ electrons both depend on $\sin^2\Theta_{\mathrm{T}}$ as

$$
\mathcal{P}_{d_{\pm 1}} \sim \frac{|R_{ps}|^2}{30} \sin^2\Theta_{\mathrm{T}}. \qquad \text{(S13)}
$$

neon and argon For neon an argon, the XUV matrix element is given by

$$
I_z = \sum_{l,m} (-i)^l e^{-i\sigma_l} k \, Y_{lm}(\theta_k, \phi_k) \, \langle Y_{lm} | \cos\theta_r | Y_{1m_{\mathrm{i}}} \rangle \int r \mathcal{F}_l(\eta, kr) \left[\frac{d}{dr} + \frac{2 - l(l+1)}{2r} \right] f_1(r) \, dr, \qquad \text{(S14)}
$$

where $f_1(r)$ is an initial p orbital. The angular integral is given by

$$\begin{aligned}\langle Y_{lm}|\cos\theta_r|Y_{1m_\mathrm{i}}\rangle &= \left\langle Y_{lm}\left|\sqrt{\frac{4\pi}{3}}Y_{10}\right|Y_{1m_\mathrm{i}}\right\rangle \\ &= \frac{1}{\sqrt{3}}\delta_{l0}\delta_{m_\mathrm{i}0} + \frac{2}{\sqrt{15}}\delta_{l2}\delta_{m_\mathrm{i}0} + \frac{1}{\sqrt{5}}\delta_{l2}(\delta_{m_\mathrm{i}-1}+\delta_{m_\mathrm{i}+1}),\end{aligned} \tag{S15}$$

and so the $s, d_0, d_{\pm1}$ intermediate state contribute, so that Eq. (S14) becomes

$$I_z = e^{-i\sigma_0}\frac{R_{sp}}{\sqrt{3}}Y_{00}(\theta_k,\phi_k) - e^{-i\sigma_2}\frac{2R_{dp}}{\sqrt{15}}Y_{20}(\theta_k,\phi_k) - e^{-i\sigma_2}\frac{R_{dp}}{\sqrt{5}}\left[Y_{2-1}(\theta_k,\phi_k)+Y_{2+1}(\theta_k,\phi_k)\right], \tag{S16}$$

where

$$R_{sp} = \int kr\mathcal{F}_0(\eta,kr)\left[\frac{d}{dr}+\frac{1}{r}\right]f_1(r)dr, \tag{S17}$$

and

$$R_{dp} = \int kr\mathcal{F}_2(\eta,kr)\left[\frac{d}{dr}-\frac{2}{r}\right]f_1(r)dr. \tag{S18}$$

Using Eq. (S16), the amplitudes in Eq. (S5) are given by

$$\begin{aligned}S^{(n=\pm1)} \sim &-2\pi i e^{-i(\phi_{2q\mp1}\pm\phi_L)}\left[\cos\Theta_\mathrm{T}\cos\theta_k + \sin\Theta_\mathrm{T}\sin\theta_k\sin\phi_k\right] \\ &\times\left\{\delta_{m_\mathrm{i}0}\left[e^{-i\sigma_0}\frac{R_{sp}}{\sqrt{3}}Y_{00}(\theta_k,\phi_k) - e^{-i\sigma_2}R_{dp}\frac{2}{\sqrt{15}}Y_{20}(\theta_k,\phi_k)\right]\right. \\ &\left.-\,\delta_{m_\mathrm{i}-1}\,e^{-i\sigma_2}\frac{R_{dp}}{\sqrt{5}}Y_{2-1}(\theta_k,\phi_k) - \delta_{m_\mathrm{i}+1}\,e^{-i\sigma_2}\frac{R_{dp}}{\sqrt{5}}Y_{2+1}(\theta_k,\phi_k)\right\}.\end{aligned} \tag{S19}$$

Expressing $\cos\theta_k$ and $\sin\theta_k\sin\phi_k$ as spherical harmonics, and then contracting the resulting

products of spherical harmonics, Eq. (S19) may be written as

$$
\begin{aligned}
S^{(\pm 1)} \sim -2\pi i e^{-i(\phi_{2q\mp 1}\pm\phi_L)} \cos\Theta_{\mathrm{T}} \Bigg\{ & \delta_{m_{\mathrm{i}}0} \left[\left(e^{-i\sigma_0} \frac{R_{sp}}{3} - e^{-i\sigma_2} \frac{4R_{dp}}{15} \right) Y_{10} - e^{-i\sigma_2} \frac{6R_{dp}}{\sqrt{525}} Y_{30} \right] \\
& - \delta_{m_{\mathrm{i}}-1} \left[e^{-i\sigma_2} R_{dp} \left(\frac{1}{5} Y_{1-1} + \sqrt{\frac{8}{175}} Y_{3-1} \right) \right] \\
& - \delta_{m_{\mathrm{i}}+1} \left[e^{-i\sigma_2} R_{dp} \left(\frac{1}{5} Y_{11} + \sqrt{\frac{8}{175}} Y_{31} \right) \right] \Bigg\} \\
-2\pi i e^{-i(\phi_{2q\mp 1}\pm\phi_L)} \sin\Theta_{\mathrm{T}} \Bigg\{ & \delta_{m_{\mathrm{i}}0} \left[\left(e^{-i\sigma_0} \frac{iR_{sp}}{\sqrt{18}} + e^{-i\sigma_2} \frac{2iR_{dp}}{\sqrt{450}} \right) (Y_{11} + Y_{1-1}) - e^{-i\sigma_2} i R_{dp} \sqrt{\frac{4}{175}} (Y_{31} + Y_{3-1}) \right] \\
& + \delta_{m_{\mathrm{i}}-1} \left[-e^{-i\sigma_2} R_{dp} \left(i\sqrt{\frac{1}{35}} Y_{3-2} + i\sqrt{\frac{3}{350}} Y_{30} - i\sqrt{\frac{1}{50}} Y_{10} \right) \right] \\
& + \delta_{m_{\mathrm{i}}+1} \left[-e^{-i\sigma_2} R_{dp} \left(i\sqrt{\frac{1}{35}} Y_{32} + i\sqrt{\frac{3}{350}} Y_{30} - i\sqrt{\frac{1}{50}} Y_{10} \right) \right] \Bigg\},
\end{aligned}
\tag{S20}
$$

From Eq. (S20), the Θ_{T}-dependent yield of f electrons is found by squaring the coefficients of the spherical harmonics Y_{3m} for $m = 0, \pm 1, \pm 2$. Note that contributions from initial states with different magnetic quantum number m_{i} must be summed incoherently. Beginning with f_0, the amplitude contains a term with coefficient $\cos\Theta_{\mathrm{T}}$ originating from an initial $m_{\mathrm{i}} = 0$, and a term with coefficient $\sin\Theta_{\mathrm{T}}$ from initial states with $m_{\mathrm{i}} = +1$ and $m_{\mathrm{i}} = -1$. These are squared and summed incoherently to give the yield of f_0 electrons, $\mathcal{P}_{f_0}$:

$$
\begin{aligned}
\mathcal{P}_{f_0} &\sim \underbrace{\frac{36}{525} |R_{dp}|^2 \cos^2\Theta_{\mathrm{T}}}_{\text{from } m_{\mathrm{i}} = 0} + \underbrace{\frac{3}{350} |R_{dp}|^2 \sin^2\Theta_{\mathrm{T}}}_{\text{from } m_{\mathrm{i}} = -1} + \underbrace{\frac{3}{350} |R_{dp}|^2 \sin^2\Theta_{\mathrm{T}}}_{\text{from } m_{\mathrm{i}} = +1}, \\
&\sim \frac{12}{175} |R_{dp}|^2 \cos^2\Theta_T + \frac{3}{175} |R_{dp}|^2 \sin^2\Theta_{\mathrm{T}}.
\end{aligned}
\tag{S21}
$$

Continuing this procedure for all f electrons in the final state, we find that

$$\mathcal{P}_{f_0} \sim \frac{3|R_{dp}|^2}{175}\left[4\cos^2\Theta_{\mathrm{T}} + \sin^2\Theta_{\mathrm{T}}\right], \tag{S22}$$

$$\mathcal{P}_{f_{\pm1}} \sim \frac{4|R_{dp}|^2}{175}\left[2\cos^2\Theta_{\mathrm{T}} + \sin^2\Theta_{\mathrm{T}}\right], \tag{S23}$$

$$\mathcal{P}_{f_{\pm2}} \sim \frac{|R_{dp}|^2}{35}\sin^2\Theta_{\mathrm{T}}. \tag{S24}$$

The yield of p_0 and $p_{\pm1}$ electrons is more complicated, and involves the relative contributions of the $p \to s \to p$ and $p \to d \to p$ pathways. Once again summing over different m_i values incoherently, their yields are given by

$$\mathcal{P}_{p_0} \sim \left|e^{-i\sigma_0}\frac{R_{sp}}{3} - e^{-i\sigma_2}\frac{4R_{dp}}{15}\right|^2\cos^2\Theta_{\mathrm{T}} + \frac{|R_{dp}|^2}{25}\sin^2\Theta_{\mathrm{T}}, \tag{S25}$$

and

$$\mathcal{P}_{p_1} \sim \frac{|R_{dp}|^2}{25}\cos^2\Theta_{\mathrm{T}} + \left|e^{-i\sigma_0}\frac{iR_{sp}}{\sqrt{18}} + e^{-i\sigma_2}\frac{2iR_{dp}}{\sqrt{450}}\right|^2\sin^2\Theta_{\mathrm{T}}. \tag{S26}$$

To determine the yields of p_0 and p_1 electrons, the coefficients of $\cos^2\Theta_{\mathrm{T}}$ and $\sin^2\Theta_{\mathrm{T}}$ are given by the photoelectron lm-resolved yields at $\Theta_{\mathrm{T}} = 0°$ and $\Theta_{\mathrm{T}} = 90°$ respectively. We obtain these yields from the RMT calculations, by integrating the lm-resolved momentum distributions over the photoelectron momentum and angular variables.

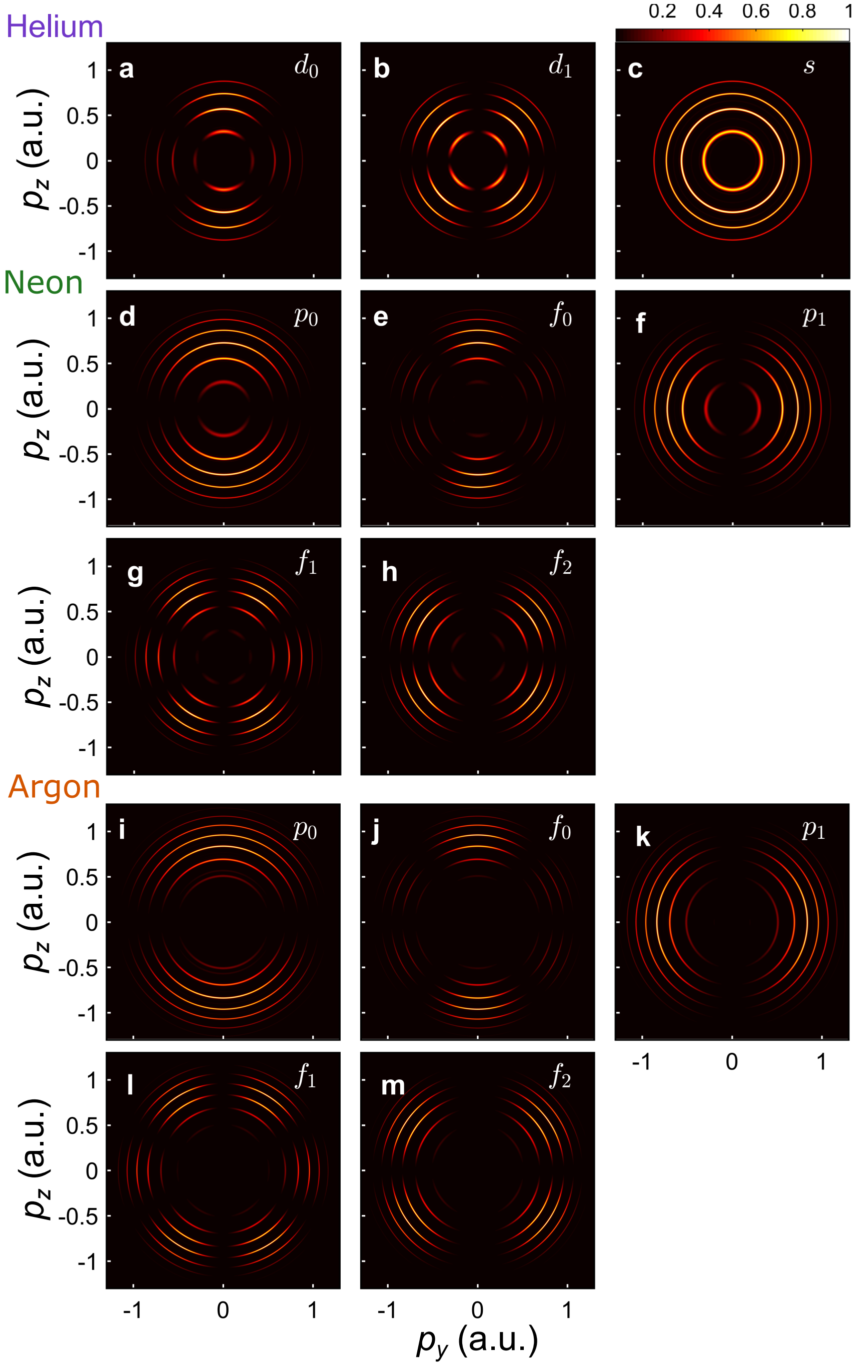

Supplementary Fig. 6: Theoretical m-resolved single partial wave PADs. (**a-c**) The single partial wave PADs of helium with (**a**) d_0, (**b**) d_1 and (**c**) s. (**d-h**) The single partial wave PADs of neon. The partial wave in each case is (**d**) p_0, (**e**) f_0, (**f**) p_1, (**g**) f_1 and (**h**) f_2. (**i-m**) As same as (**d-h**) but for the argon atoms. The m-resolved single partial wave PADs are averaged over pump-probe time delays.

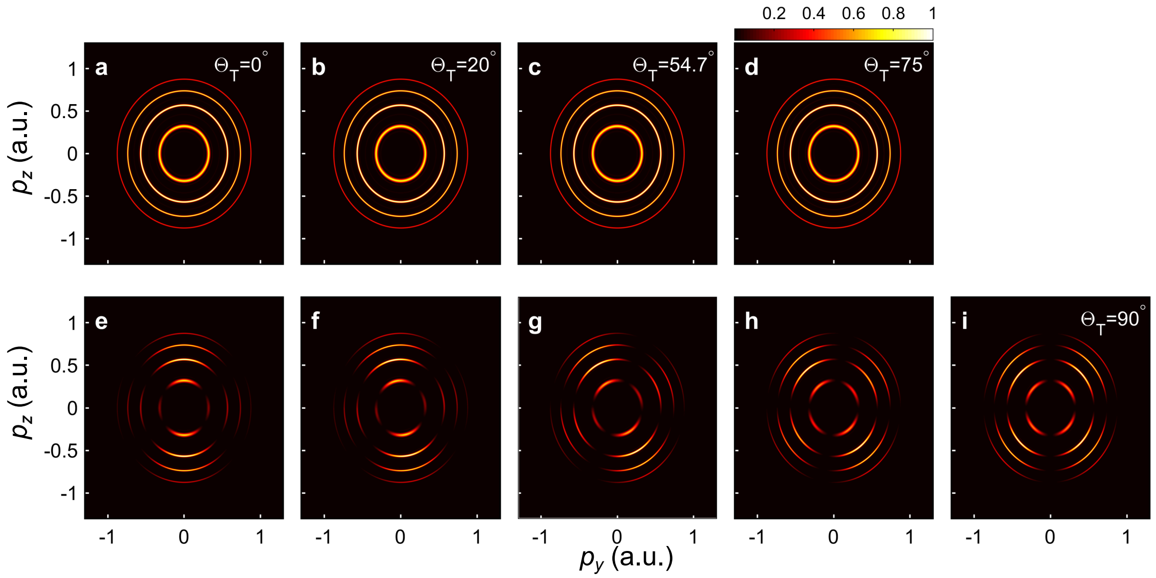

Supplementary Fig. 7: Theoretical l-resolved partial wave PADs of helium. (**a-d**) The s-wave PADs of helium averaged over pump-probe time delays as a function of the skew angle, where the Θ_{T} = (**a**) $0°$, (**b**) $20°$, (**c**) $54.7°$ and (**d**) $75°$. (**e-i**) The d-wave PADs of helium. The skew angle conditions of (**e-h**) are as the same as (**a-d**) and $\Theta_{\mathrm{T}} = 90°$ in (**i**).

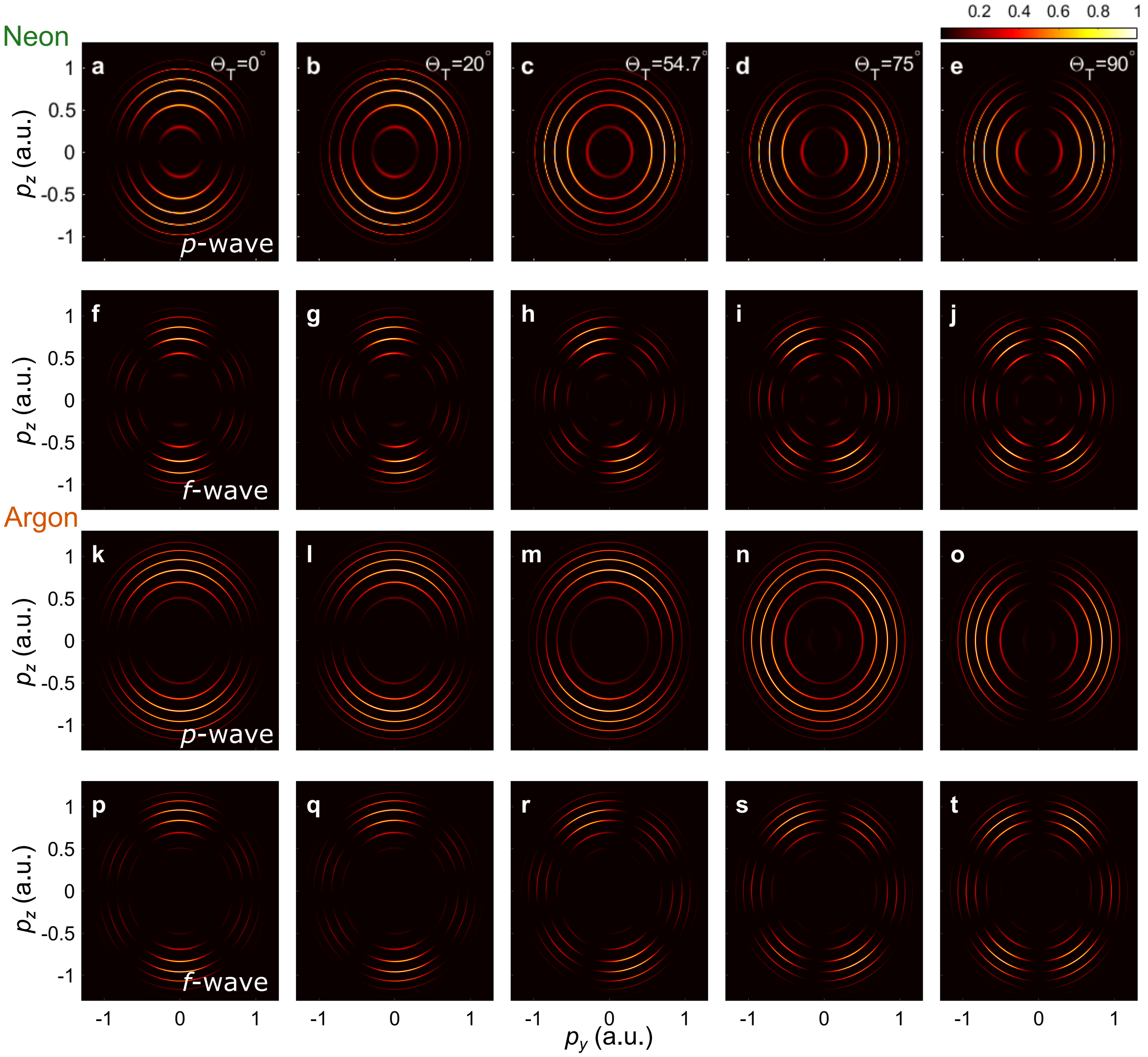

Supplementary Fig. 8: Theoretical l-resolved partial wave PADs with the P_0 residual-ion state. (**a-e**) The p-wave PADs of neon averaged over pump-probe time delays as a function of the skew angle, where the $\Theta_\mathrm{T} =$ (**a**) $0°$, (**b**) $20°$, (**c**) $54.7°$, (**d**) $75°$ and (**e**) $90°$. (**f-j**) The f-wave PADs of neon as the same skew angle as (**a-e**). (**k-o**), (**p-t**) The p-and f-wave PADs of argon.

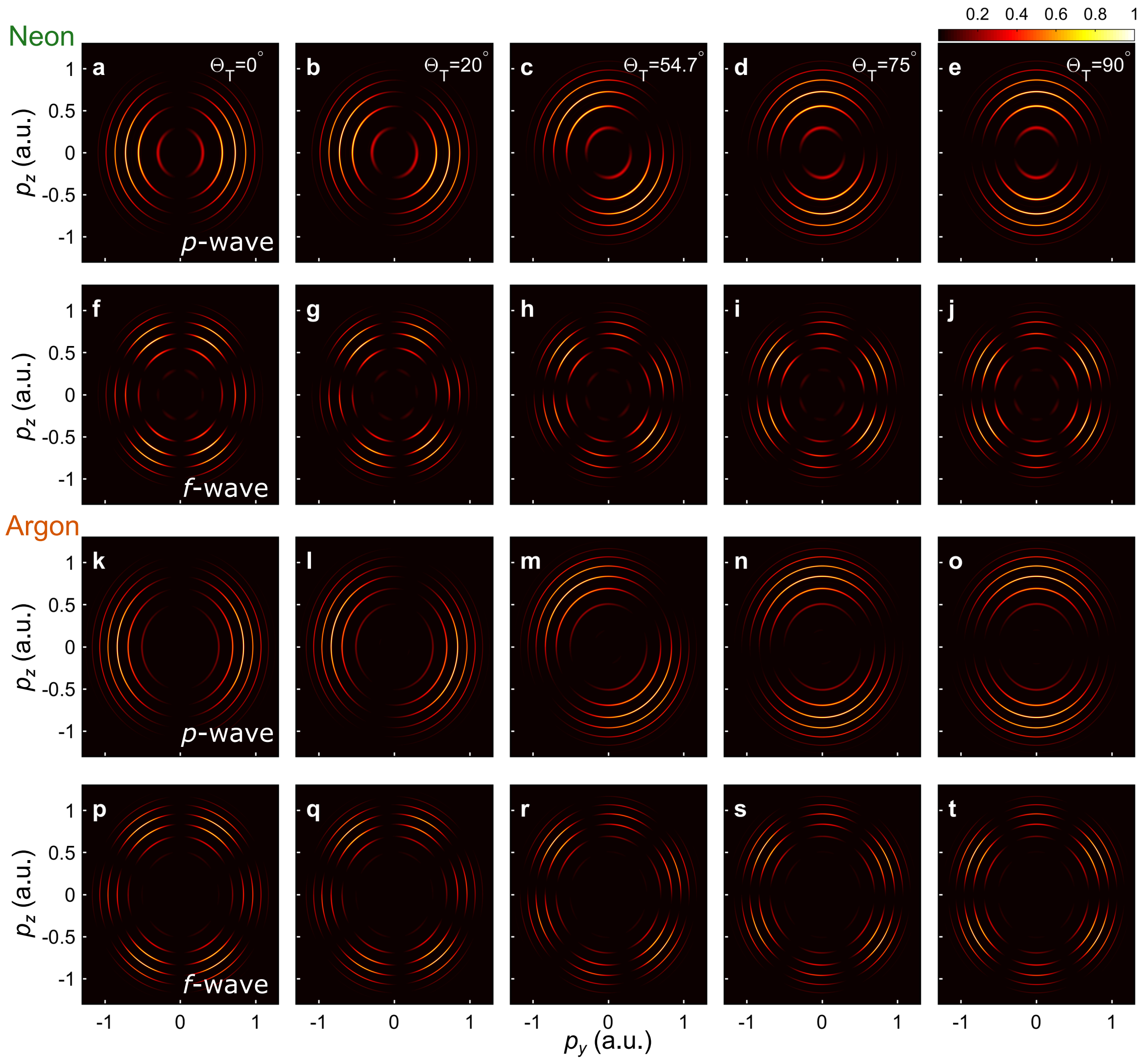

Supplementary Fig. 9: Theoretical l-resolved partial wave PADs with the $P_{\pm1}$ residual-ion state . (**a-e**) The p-wave PADs of neon averaged over pump-probe time delays as a function of the skew angle. (**f-j**) The f-wave PADs of neon. (**k-o**), (**p-t**) The p- and f-wave PADs of argon. The skew angles are the same as those in Supplementary Figs. 8.

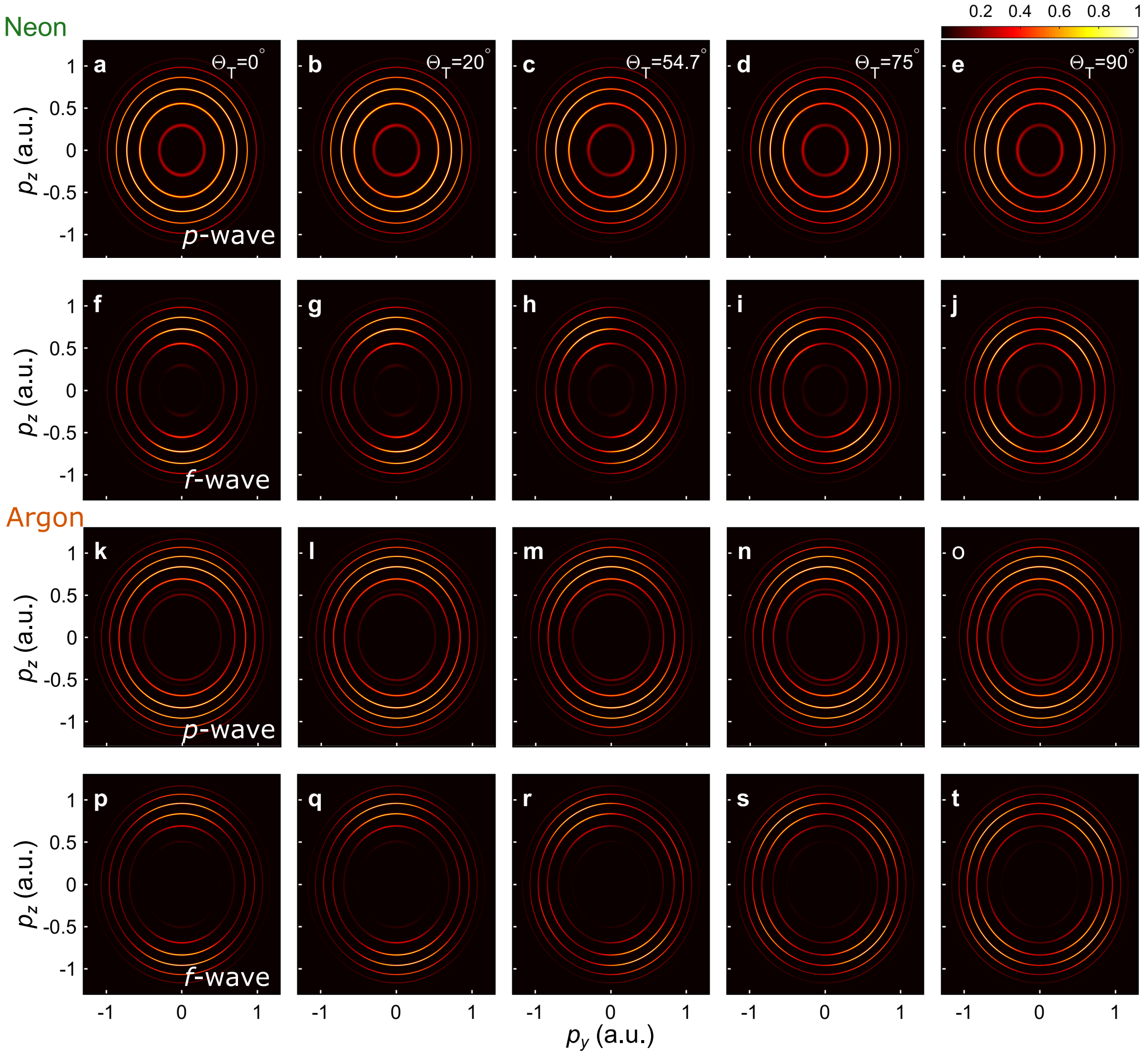

Supplementary Fig. 10: Theoretical l-resolved partial wave PADs with the incoherent sum of P_0 and $P_{\pm1}$ residual ion states. (**a-e**) The p-wave PADs of neon averaged over pump-probe time delays as a function of the skew angle. (**f-j**) The f-wave PADs of neon. (**k-o**), (**p-t**) The p- and f-wave PADs of argon. The skew angles are the same as those in Supplementary Figs. 8.

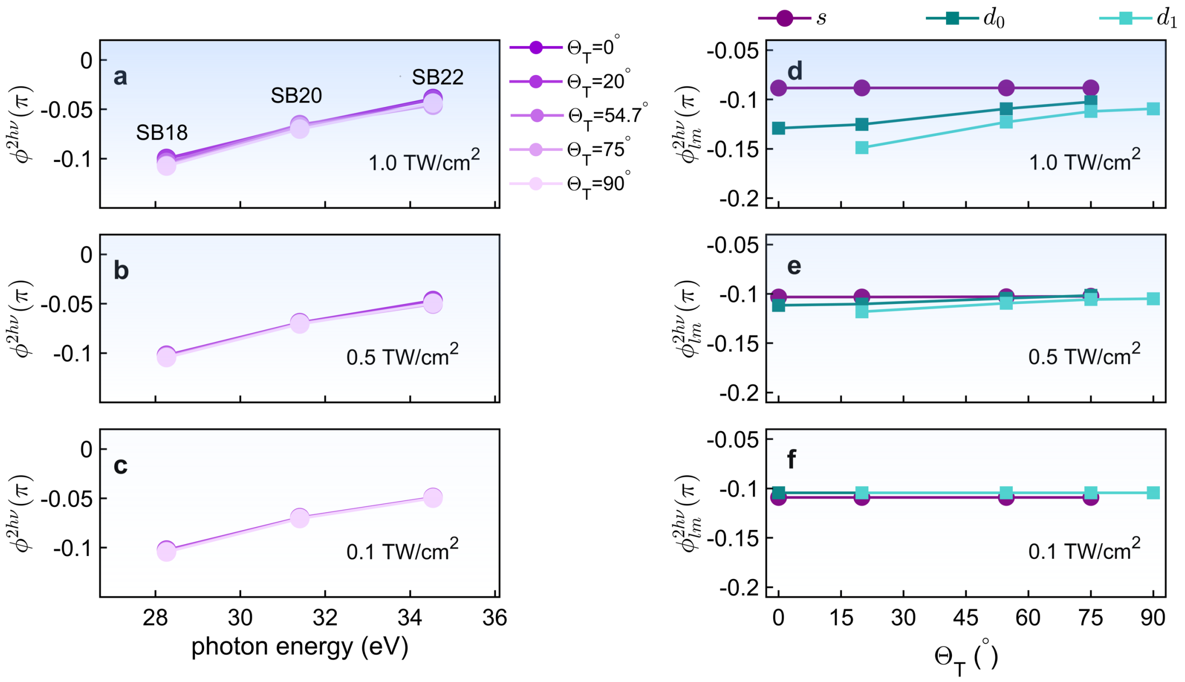

Supplementary Fig. 11: Atomic phase shifts of helium as a function of NIR intensity and the skew angle. (**a-c**) The theoretical effective phase shifts averaged over the photoelectron emission angle where the NIR field intensity is setting at (**a**) $1.0\mathrm{TW/cm}^2$, (**b**) $0.5\mathrm{TW/cm}^2$ and (**c**) $0.1\mathrm{TW/cm}^2$. The skew angle condition shown by solid circles with colors from dark purple to light purple is $0°$, $20°$, $54.7°$, $75°$ and $90°$, respectively. (**d-f**) The theoretical partial wave phase shifts of helium. The purple, dark turquoise and cyan markers represent the phase shift of s-, d_0- and d_1- wave, respectively. The NIR intensities are the same as those in (**a-c**).

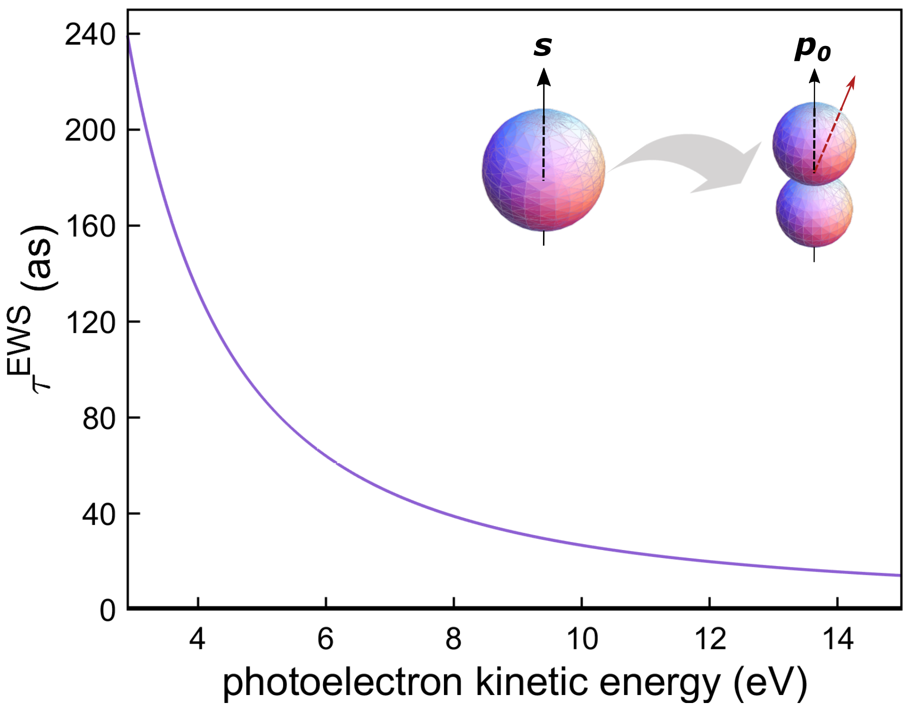

Supplementary Fig. 12: EWS time delay of helium as a function of the kinetic energy. The purple line shows the Eisenbud-Wigner-Smith time delay of helium calculated via the ePolyScat code[21,22].

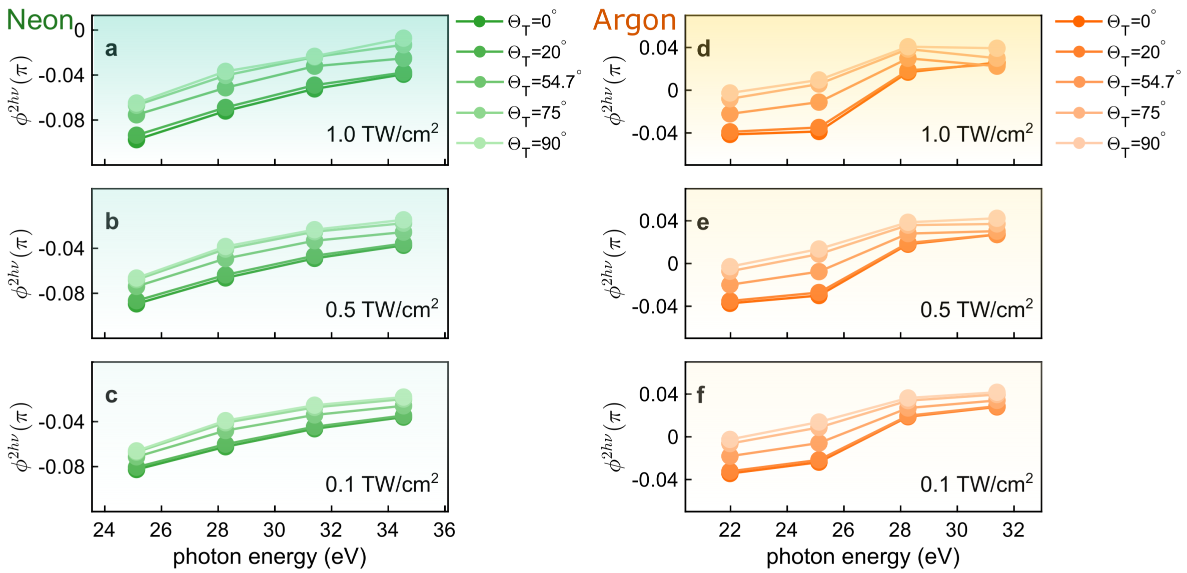

Supplementary Fig. 13: Effective phase shifts of neon and argon as a function of NIR intensity and the skew angle. (**a-c**) The theoretical effective phase shifts of neon. The selected conditions are as the same as Supplementary Figs. 11 (**a-c**) and the sidebands covering SB16 to SB22 of neon. (**d-f**) The same as (**a-c**) but for argon covering sidebands from SB14 to SB20.

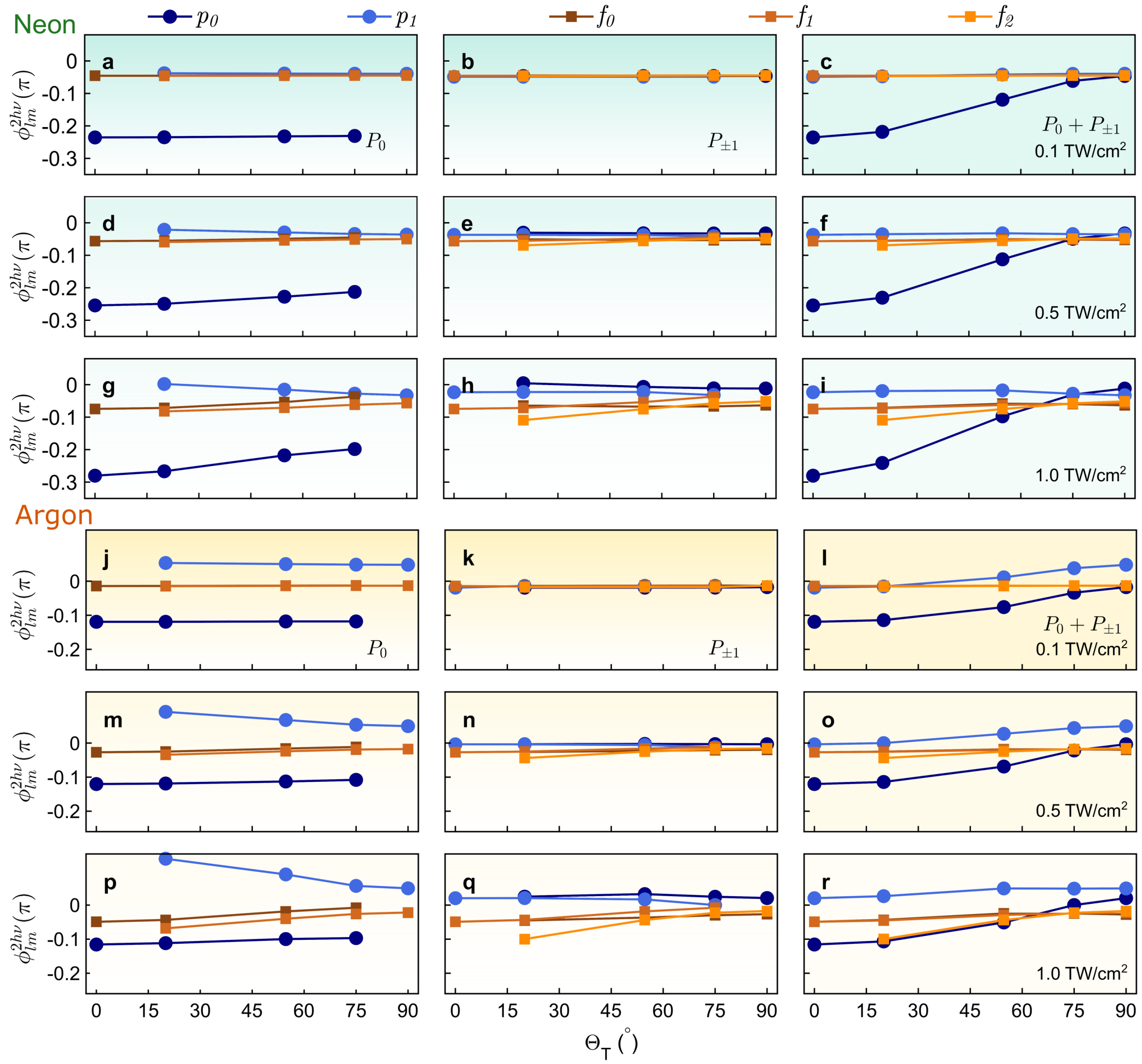

Supplementary Fig. 14: Ionic state resolved partial wave phase shifts of neon and argon as a function of NIR intensity and the skew angle. (**a-c**) The ionic state resolved partial wave phase shift of SB18 of neon, where the NIR intensity is $0.1\mathrm{TW/cm^2}$. The residual-ion state is (**a**) P_0, (**b**) $P_{\pm1}$, and (**c**) the incoherent sum of P_0 and $P_{\pm1}$. The blue solid circles and yellow squares from dark to light represent the results of $p_{0,1}$- and $f_{0,1,2}$-waves, respectively .(**d-f**) and (**g-i**) The selected ionic states are as the same as (**a-c**) with the different NIR field intensities at (**d-f**) $0.5\mathrm{TW/cm^2}$ and (**g-i**) $1.0\mathrm{TW/cm^2}$. (**j-r**) As the same as (**a-i**) but for argon.

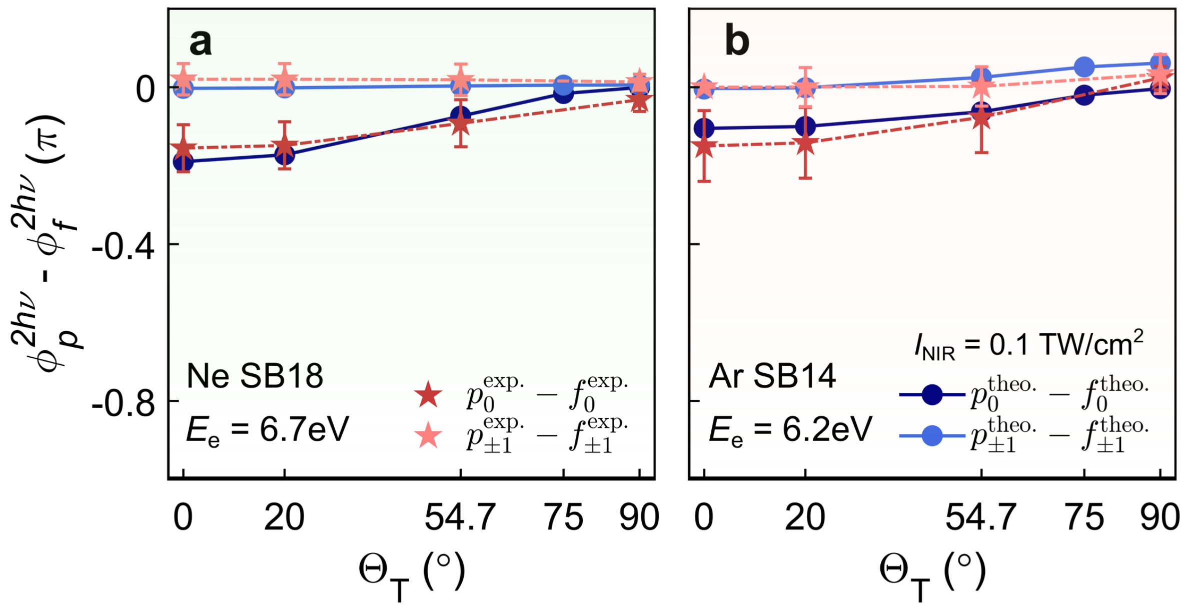

Supplementary Fig. 15: Relative partial wave resolved phase shift between p- and f-waves. (**a**) The skew-angle dependent two-photon transition phase shift between p_0/f_0 and $p_{\pm1}/f_{\pm1}$ in the neon atom at SB18. The dark and light red stars show the reconstructed $\Delta\phi^{2h\nu}_{p_0-f_0}$, $\Delta\phi^{2h\nu}_{p_1-f_1}$ from the experimental measurements by applying the partial wave proportions illustrated by the SPA model and the error bars represent the standard deviation. The blue circles show the results from theoretical simulation with the NIR intensity of $I_{\text{NIR}} = 0.1\text{TW/cm}^2$. (**b**) As the same as (**b**) but for argon atoms.

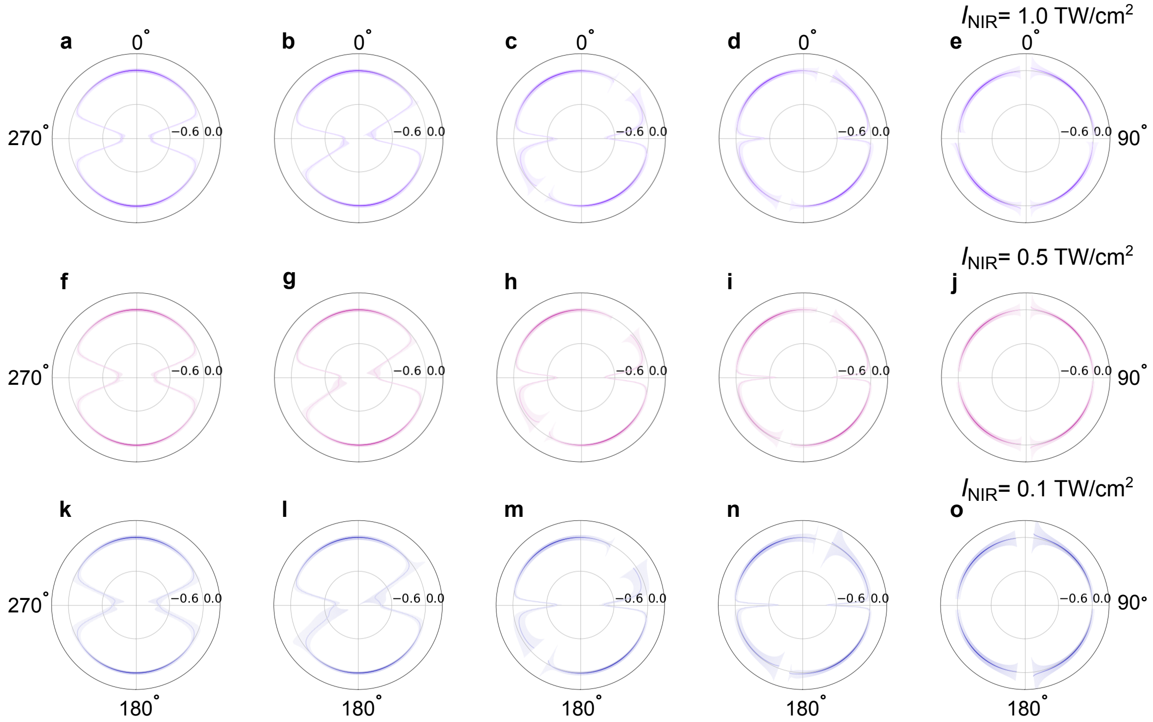

Supplementary Fig. 16: Angle-resolved phase shifts of helium as a function of NIR intensity and the skew angle. (**a-e**) The angle-resolved relative atomic phase shift of SB18 of helium, where the NIR intensity is $1.0\mathrm{TW/cm^2}$. The skew angle in each case is (**a**) $0°$, (**b**) $20°$, (**c**) $54.7°$, (**d**) $75°$ and (**d**) $90°$, respectively. (**f-j**) and (**k-o**) The angle-resolved relative atomic phase shift of SB18 of helium. The skew angle conditions are as the same as (**a-e**) with the different NIR field intensities at (**f-j**) $0.5\mathrm{TW/cm^2}$ and (**k-o**) $0.1\mathrm{TW/cm^2}$.

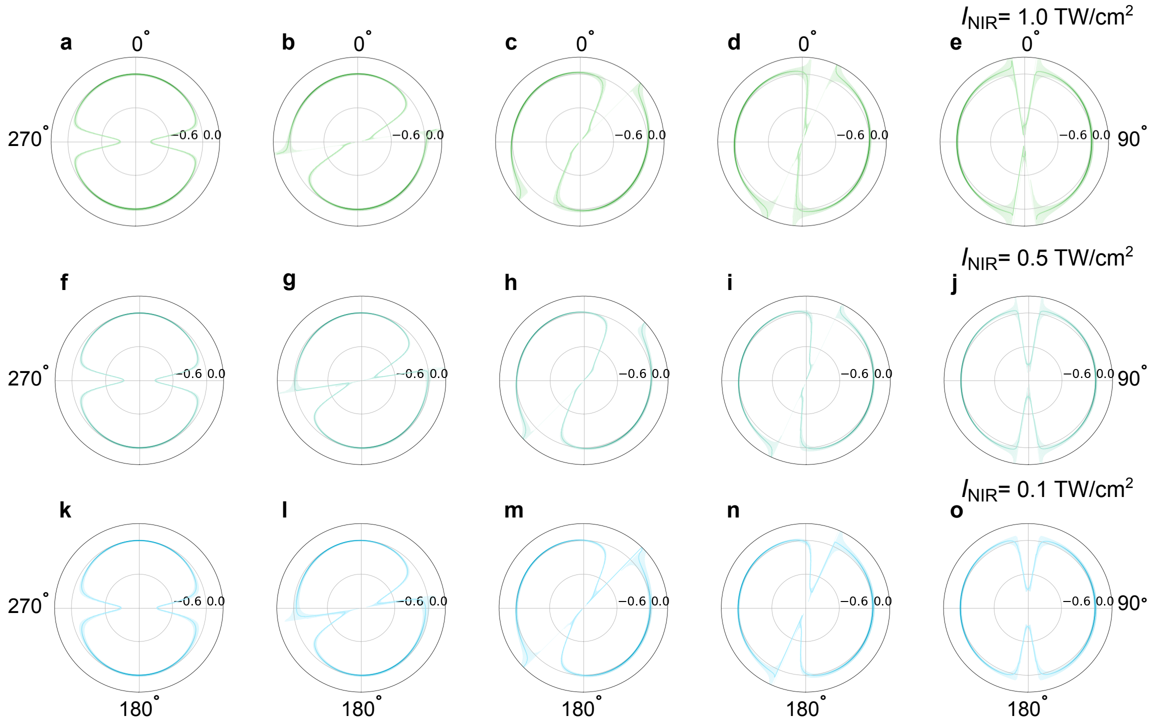

Supplementary Fig. 17: Angle-resolved phase shifts of neon as a function of NIR intensity and the skew angle. (**a-e**) The angle-resolved relative atomic phase shift of SB18 of neon, where the NIR intensity is $1.0\mathrm{TW/cm^2}$. (**f-j**) and (**k-o**) The angle-resolved relative atomic phase shifts of SB18 of neon with the different NIR field intensities at (**f-j**) $0.5\mathrm{TW/cm^2}$ and (**k-o**) $0.1\mathrm{TW/cm^2}$. The skew angle conditions are as the same as Supplementary Figs. 16.

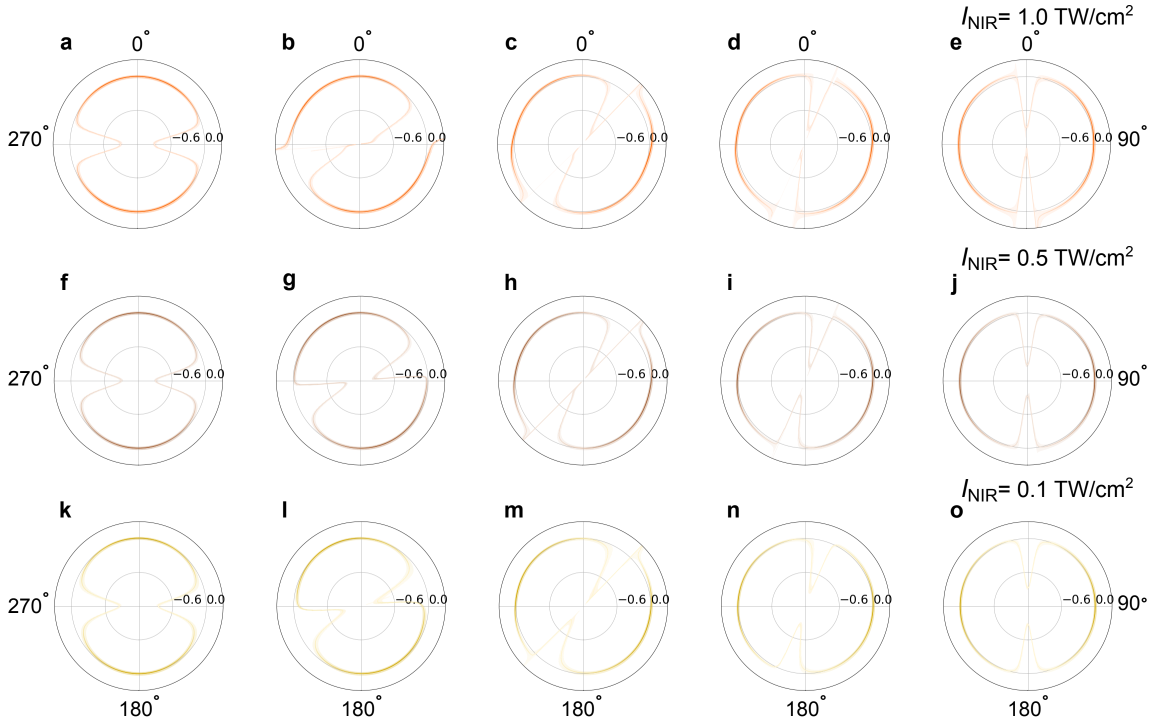

Supplementary Fig. 18: Angle-resolved phase shifts of argon as a function of NIR intensity and the skew angle. (**a-e**) The angle-resolved relative atomic phase shift of SB14 of argon, where the NIR intensity is $1.0\mathrm{TW/cm^2}$. (**f-j**) and (**k-o**) The angle-resolved relative atomic phase shifts of SB14 of argon with the different NIR field intensities at (**f-j**) $0.5\mathrm{TW/cm^2}$ and (**k-o**) $0.1\mathrm{TW/cm^2}$. The skew angle conditions are as the same as Supplementary Figs. 16.

Supplementary References

1. Gagnon, J., Goulielmakis, E. & Yakovlev, V. S. The accurate FROG characterization of attosecond pulses from streaking measurements. *Applied Physics B: Lasers and Optics* **92**, 25–32 (2008).

2. Amusia, M. Y., Cherepkov, N. A. & Chernysheva, L. V. Angular distribution of photoelectrons with many-electron correlations. *Physics Letters* **40A**, 15–16 (1972).

3. Taylor, K. T. Photoelectron angular-distribution beta parameters for neon and argon. *Journal of Physics B* **10**, L699 (1977).

4. Codling, K., Houlgate, R. G., West, J. B. & Woodruff, P. R. Angular distribution and photoionization measurements on the 2p and 2s electrons in neon. *Journal of Physics B* **9**, L83 (1976).

5. Mondal, S. *et al.* Pulse-delay effects in the angular distribution of near-threshold EUV + IR two-photon ionization of Ne. *Physical Review A* **89**, 013415 (2014).

6. Ishikawa, K. L., Kazansky, A. K., Kabachnik, N. M. & Ueda, K. Theoretical study of pulse delay effects in the photoelectron angular distribution of near-threshold EUV+IR two-photon ionization of atoms. *Physical Review A* **90**, 023408 (2014).

7. Swoboda, M. *et al.* Phase measurement of resonant two-photon ionization in helium. *Physical Review Letters* **104**, 103003 (2010).

8. Dahlström, J. M. *et al.* Theory of attosecond delays in laser-assisted photoionization. *Chemical Physics* **414**, 53–64 (2013).

9. Dahlström, J. M., L'Huillier, A. & Maquet, A. Introduction to attosecond delays in photoionization. *Journal of Physics B* **45**, 183001 (2012).

10. Fuchs, J. *et al.* Time delays from one-photon transitions in the continuum. *Optica* **7**, 154–161 (2020).

11. Ivanov, I. A. & Kheifets, A. S. Angle-dependent time delay in two-color XUV+IR photoemission of He and Ne. *Physical Review A* **96**, 013408 (2017).

12. Cirelli, C. *et al.* Anisotropic photoemission time delays close to a Fano resonance. *Nature Communications* **9**, 955 (2018).

13. Joseph, J. *et al.* Angle-resolved studies of XUV-IR two-photon ionization in the RABBITT scheme. *Journal of Physics B* **53**, 184007 (2020).

14. Moore, L. R. *et al.* The RMT method for many-electron atomic systems in intense short-pulse laser light. *Journal of Modern Optics* **58**, 1132 (2011).

15. Clarke, D. D. A., Armstrong, G. S. J., Brown, A. C. & van der Hart, H. W. r-matrix-with-time-dependence theory for ultrafast atomic processes in arbitrary light fields. *Physical Review A* **98**, 053442 (2018).

16. Brown, A. C. *et al.* RMT: R-matrix with time-dependence. solving the semi-relativistic, time-dependent schrödinger equation for general, multielectron atoms and molecules in intense, ultrashort, arbitrarily polarized laser pulses. *Computer Physics Communications* **250**, 107062 (2020).

17. Burke, P. G. & Taylor, K. T. R-matrix theory of photoionization. Application to neon and argon. *Journal of Physics B* **8**, 2620 (1975).

18. Brown, A. C., Robinson, D. J. & van der Hart, H. W. Atomic harmonic generation in time-dependent R-matrix theory. *Phys. Rev. A* **86**, 053420 (2012).

19. van der Hart, H. W., Lysaght, M. A. & Burke, P. Momentum distributions of electrons ejected during ultrashort laser interactions with multielectron atoms described using the r-matrix basis sets. *Physical Review A* **77**, 065401 (2008).

20. Toma, E. S. & Muller, H. G. Calculation of matrix elements for mixed extreme-ultraviolet-infrared two-photon above-threshold ionization of argon. *Journal of Physics B* **35**, 3435–3442 (2002).

21. Natalense, A. P. Cross section and asymmetry parameter calculation for sulfur 1 s photoionization of SF6. *Journal of Chemical Physics* **111**, 5344–5348 (1999).

22. Gianturco, F. A., Lucchese, R. R. & Sanna, N. Calculation of low-energy elastic cross sections for electron-CF 4 scattering. *The Journal of Chemical Physics* **100**, 6464–6471 (1994).

23. Kennedy, D. J. & Manson, S. T. Photoionization of the noble gases: Cross sections and angular distributions. *Physical Review A* **5**, 227–247 (1972).

24. Maquet, A. & Taïeb, R. Two-colour IR+XUV spectroscopies: The ”soft-photon approximation”. *Journal of Modern Optics* **54**, 1847–1857 (2007).
